# Supplementary material for: Allosteric regulation and crystallographic fragment screening of SARS-CoV-2 NSP15 endoribonuclease
Source: Nucleic Acids Res. 2023 Apr 28;51(10):5255–70. doi: 10.1093/nar/gkad314 (PMC10250223; doi:10.1093/nar/gkad314)
Supplement: gkad314_Supplemental_Files [file gkad314_supplemental_files.zip › Supplementary datasets.zip!/183/Refine_4/01-BUSTER/Cycle-4/LIST.html]

Refining

**BUSTER 2.X**: A suite of programs for macromolecular structure refinement by maximum-likelihood and Bayesian methods  
Copyright © 1992-2020 Global Phasing Ltd.  
All rights reserved.  
Authors: G. Bricogne, E. Blanc, M. Brandl, C. Flensburg, P. Keller, W. Paciorek, P. Roversi, O. Smart, C. Vonrhein, T. Womack  

Please reference use of the software as:

- BUSTER-TNT 2.X, Global Phasing Ltd, Sheraton House, Cambridge CB3 0AX, UK

Version: 2.10.3 for linux64-ifort made on May 20 2020 at 01:14:34  
Licenced to: Diamond Light Source, Scientific Software  

```
---


### Parsing of the BUSTER cardfile

TITLE : autoBUSTER refinement

   Cell information

   Symmetry information

 Using CCP4 X-ray form factors.

   Missing structure chemical composition

 Using ATOM block for chemical composition.


   Shell information

### Parsing of the TNT cardfile


---


### Reading observations file

 Number of reflexions in input:  70521

      Number of accepted reflexions:  64732

      Number of rejected reflexions:      0

   Reflexions in test set:            3243

   Reflexions in working set:        61489


---


### 1. Generating initial model

 2.10 * Shannon sampling rate. NX NY NZ:   288  288  240


  Effective Shannon rates are:    2.15    2.15    2.45


   Allocated BUFFER of size:     76.465 Mb.

   Initial Geometry Information


 No missing atoms declared


   Summary of model volumes

 No missing atoms declared: no prior computed


 Computing bulk solvent model scattering


 NCSMASK PDB radius for solvent mask:     2.15 Angstrom

 running PDBSET (setting cell and spacegroup P1) ... done
 running NCSMASK [1] (creating mask, SMOOTH 1.04563) ... done
 running MAPDUMP (to check if reduction to unit cell is needed) ... done
 running MAPMASK (converting mask to map) ... done

   Generation of babslv by theta-filter

   Electron density in frg mask:  0.420 Å-1

 FOBS  put on rough absolute scale by Wilson plot :
           K        =  0.49530E+02
           B        =     -7.160

 

### Cycle 0001: MaxLik scaling and refinement


   MaxLik Scaling Cycle 0001

   Wilson plot

   Statistics in resolution bins just after scaling
```

|  |  |
| --- | --- |
|  | - Rxpct factors plot- Rxpct factors table- <Log-Lik Gain>- Ln Sigma A- Correlation Coefficients |

```
   <Log-Likelihood> of null-hypothesis

Structure Factor Amplitudes


   Average Figure of Merit

 Switching off refinement of overall temperature factor


 Added B =    -0.0090 Å2 to all atoms in fragment


 Added B =    -0.0090 Å2 to B_SOLV

```
gelly refinement module
 Authors (2004-2009) O. S. Smart, C. Flensburg, W. Paciorek, C. Vonrhein, 
                     M. Brandl, T. O. Womack and G. Bricogne
         (2010-2011) O. S. Smart, C. Flensburg, W. Paciorek, C. Vonrhein, 
                     T. O. Womack and G. Bricogne
         (2012-2014) O. S. Smart, C. Flensburg, W. Paciorek, C. Vonrhein, 
                     and G. Bricogne
         (2015-2020) G. Bricogne, C. Flensburg, W. Paciorek, C. Vonrhein
 Copyright © 2004-2020 Global Phasing Ltd. 
 All rights reserved.
 gelly module compilation date May 20 2020 01:11:20
 
 gelly uses routines from TNT geometry. References: 
     Tronrud, D. E., Ten Eyck, L. F., Matthews, B. W. (1987)
     "An Efficient General-Purpose Least-Squares Refinement Program 
     for Macromolecular Structures". Acta Crystallogr A, 43:489-501.
     Tronrud, D. E. (1997). "The TNT Refinement Package", 
     in Macromolecular Crystallography, Part B, Eds Charlie Carter 
     and Robert Sweet, Methods in Enzymology, 277:306-319.
 wildcard matching function by Julian Robichaux http://www.nsftools.com

 No -random_seed flag found. Random number stream initialised using:
2147483562
2147483398

 Control flags set to:  -autoncs -hydrogens_input_occ2zero \
                     -hydrogens_output_occ2heavy -screen \
                     100 -screen_sigma \
                     3.0 -keeppdb \
                     1000000 -taomonitor_verbose \
                     2 
 

 ############### Starting setup via TNT routines

 Setting up tnt routines including license check:

                   TNT Stereochemistry Program
 Version: 5.22.3.0
 Licence for Diamond Light Source, Scientific Software is valid
 
 Using TNT geometry routines to load the tnt control file: gelly.tnt
 Using TNT routines to load initial coordinates from file fragment.cor
 Expecting 6125 atoms

 Found     2202 "NOTE BUSTER_RESIDUE_ATOM_DATA" cards
 Have copied information for    6125 atoms from TNT to gelly's store 
 
 The TNT WEIGHT cards used by this run:
 -----WEIGHT       BOND     2.0
 -----WEIGHT       ANGLE    2.0
 -----WEIGHT       IMPROPER 0.0
 -----WEIGHT       TORSION  2.0
 -----WEIGHT       PSEUDO   0.0
 -----WEIGHT       TRIGONAL 2.0
 -----WEIGHT       PLANE    5.0
 -----WEIGHT       CONTACT  5.0
 -----WEIGHT       BCORREL  10.0
 -----WEIGHT       CHIRAL   5.0
 -----WEIGHT       NCS      50.0
 -----WEIGHT       IDEAL    4.0
 -----WEIGHT       RFACTOR  6.45
 
 Dictionary versions picked up from NOTE BUSTER_RESTRAINT_DICT cards as: 
 ------ protgeo_eh99.dat (V1.6.4.17) 20191001  STANDARD AMINO ACID DICTIONARY. BONDS AND ANGLES FROM ENGH AND HUBER EH99. OTHER VALUES BASED ON PREVIOUS TNT OR TAKEN FROM CCP4. INCLUDES HYDROGEN ATOMS.
 ------ exoticaa.dat (V1.3.4.15) 20200327  COLLECTION OF NON-STANDARD AMINO ACIDS, MAINLY EH91 WITHOUT IDEAL DISTANCE INFO
 ------ nuclgeo.dat (V1.13.4.8) 20190909
 ------ bcorrel.dat (V1.15.10.2) 20190616
 ------ contact.dat (V1.15.12.10) 20191121
 ------ idealdist_contact.dat (V1.3.4.12) 20191001  IDEAL-DISTANCE CONTACT TERM DATA AS USED IN PROLSQ. VALUES USED HERE ARE BASED ON DATA HARVESTING OF PDB AS OF 20190109.
 ------ restraints for LIG (Unknown) from cif dictionary Z2856434903.cif
 ------ restraints for CIT (CITRIC ACID) from cif dictionary CIT.cif; generated by GRADE_PDB_LIGAND 1.2.12 (October 4 2016) using MOGUL 1.7.2(RC1), CSD as538be, with quantum mechanics RM1 ((DatabaseID PDB-CIT))
 ------ assume.dat (V1.9.4.2) 20180705
 
 Have picked up spacegroup name for pdb output as 'P 63      '
 Setting up Chain/Residue arrays
 
 Have command line specification -autoncs*
     Have found       1 NCS relations
     Pairwise matching residue number and type between chains: 
                    A        B        S    
          A         .       99%       -   
          B                  .        -   
          S                           .   
       Table key--                                                       
          all   : two chains have 100% match and same number of residues 
         multi  : ditto and they contribute to a multi chain ncs cluster 
          ***%  : two chains have >80% matching residue number/type      
           -    : chains appear to be unrelated                          
      chain A     have identified ncs to     1 other chains. So will weight each autoncs by (       6.666/#)=      6.6660
      chain B     have identified ncs to     1 other chains. So will weight each autoncs by (       6.666/#)=      6.6660
      chain S     have identified ncs to     0 other chains. 
     For .Gelly cards produced see below - after pruning
 
 Setting up BUSTER_SET groups
 Have found    89 BUSTER_SET cards
 Now silently applying automatic/dictionary set definitions. For a verbose listing use '-verbose_set' flag.
 Applying user defined set definitions:
 =====NOTE BUSTER_SET ncsautoXcld = Water --> set 'ncsautoXcld' now has   581 atoms 
 =====NOTE BUSTER_SET ncsautoABset = Chain_B \ ncsautoXcld --> set 'ncsautoABset' now has  2774 atoms 
 Have defined    48 sets: 
      set:                 'All' has   6125 atoms in   1281 residues - automatically defined
      set:               'Empty' has      0 atoms in      0 residues - automatically defined
      set:            'ExoticAA' has      0 atoms in      0 residues - defined by dictionary file
      set:           'ExoticNUC' has      0 atoms in      0 residues - defined by dictionary file
      set:              'NotHET' has   5514 atoms in    698 residues - defined by dictionary file
      set:             'Chain_A' has   2770 atoms in    350 residues - automatically defined
      set:             'Chain_B' has   2774 atoms in    350 residues - automatically defined
      set:             'Chain_S' has    581 atoms in    581 residues - automatically defined
      set:               'Water' has    581 atoms in    581 residues - defined by dictionary file
      set:          'StdProtein' has   5514 atoms in    698 residues - defined by dictionary file
      set:             'Protein' has   5514 atoms in    698 residues - defined by dictionary file
      set:                'Back' has   2794 atoms in    698 residues - defined by dictionary file
      set:                'Side' has   2720 atoms in    650 residues - defined by dictionary file
      set:        'RnaCanonical' has      0 atoms in      0 residues - defined by dictionary file
      set:        'DnaCanonical' has      0 atoms in      0 residues - defined by dictionary file
      set:     'RnaDnaCanonical' has      0 atoms in      0 residues - defined by dictionary file
      set:     'DnaRnaCanonical' has      0 atoms in      0 residues - defined by dictionary file
      set:         'RnaExtended' has      0 atoms in      0 residues - defined by dictionary file
      set:         'DnaExtended' has      0 atoms in      0 residues - defined by dictionary file
      set:      'RnaDnaExtended' has      0 atoms in      0 residues - defined by dictionary file
      set:      'DnaRnaExtended' has      0 atoms in      0 residues - defined by dictionary file
      set:                 'Dna' has      0 atoms in      0 residues - defined by dictionary file
      set:                 'Rna' has      0 atoms in      0 residues - defined by dictionary file
      set:              'RnaDna' has      0 atoms in      0 residues - defined by dictionary file
      set:              'DnaRna' has      0 atoms in      0 residues - defined by dictionary file
      set:             'Nucleic' has      0 atoms in      0 residues - defined by dictionary file
      set:             'Polymer' has   5514 atoms in    698 residues - defined by dictionary file
      set: 'BufferWeichenberger' has     13 atoms in      1 residues - defined by dictionary file
      set:    'BufferAdditional' has      0 atoms in      0 residues - defined by dictionary file
      set:              'Buffer' has     13 atoms in      1 residues - defined by dictionary file
      set:         'MetalAlkali' has      0 atoms in      0 residues - defined by dictionary file
      set:  'MetalAlkalineEarth' has      0 atoms in      0 residues - defined by dictionary file
      set:     'MetalTransition' has      0 atoms in      0 residues - defined by dictionary file
      set: 'MetalPostTransition' has      0 atoms in      0 residues - defined by dictionary file
      set:    'MetalLanthanides' has      0 atoms in      0 residues - defined by dictionary file
      set:               'Metal' has      0 atoms in      0 residues - defined by dictionary file
      set:         'SugarAlphaD' has      0 atoms in      0 residues - defined by dictionary file
      set:         'SugarAlphaL' has      0 atoms in      0 residues - defined by dictionary file
      set:               'Sugar' has      0 atoms in      0 residues - defined by dictionary file
      set:               'Other' has     30 atoms in      2 residues - defined by dictionary file
      set:            'Hydrogen' has      0 atoms in      0 residues - defined by dictionary file
      set:         'nonHydrogen' has   6125 atoms in   1281 residues - defined by dictionary file
      set:             'OccZero' has      0 atoms in      0 residues - defined by dictionary file
      set:          'OccNonZero' has   6125 atoms in   1281 residues - defined by dictionary file
      set:             'OccFull' has   6076 atoms in   1278 residues - defined by dictionary file
      set:          'OccNonFull' has     49 atoms in      3 residues - defined by dictionary file
      set:         'ncsautoXcld' has    581 atoms in    581 residues - user specified
      set:        'ncsautoABset' has   2774 atoms in    350 residues - user specified
 No command line arguments '-occ_allhet' or '-occ_restyp' so will not setup automated occcupancy refinement
 
 Interpreting CONSTANT and FREE cards: 
 =====CONSTANT     OCC
 Have found and interpreted      1 CONSTANT cards in the TNT input. 
 Before any COMBINE - Number of atoms with fixed XYZ =     0
 Before any COMBINE - Number of atoms with fixed B   =     0
 Before any COMBINE - Number of atoms with fixed OCC =  6125
 Before any COMBINE - Number of atoms with fixed BIJ =   581
                            cf total number of atoms =  6125
 
 There are no COMBINE cards to be found in the TNT card input 
 
 There are no NOTE BUSTER_COMBINE_RES cards to be found in the TNT card input 

 ############### Working out full connectivity list


 ############### Working out equivalent atoms


 Residue with most atoms given below:
        B|401:C01(LIG)      
        B|401:N02(LIG)      
        B|401:C03(LIG)      
        B|401:C04(LIG)      
        B|401:N05(LIG)      
        B|401:C06(LIG)      
        B|401:C07(LIG)      
        B|401:C08(LIG)      
        B|401:C09(LIG)      
        B|401:C10(LIG)      
        B|401:C11(LIG)      
        B|401:N12(LIG)      
        B|401:C13(LIG)      
        B|401:O14(LIG)      
        B|401:C15(LIG)      
        B|401:C16(LIG)      
        B|401:C17(LIG)      


 Have no '-equiv_check_plus' argument - so will take atom type into
 account when checking for equivalence of otherwise identical atoms.
 

 ############### Looking at dihedrals


 ############### Looking at chirals

 NOTE BUSTER_CHIRAL_ANGLECUT card found - read value=    -31.00 degs from it
 NOTE BUSTER_CHIRAL_SIGMA card found - read value=      1.70 degs from it
 RANGE allowed for B variables in refinement:   from      3.00000 to    300.00000

 ############### Preparing for PDB output


 ############### Processing extra restraints explicitly defined

 No 'NOTE BUSTER_TARGET' cards or -target?? args found (used to read coordinates files for soft ncs to external)
 No 'NOTE BUSTER_OCCSUM'     cards found - so not using sum of occupancy restraints
 No 'NOTE BUSTER_DISTANCE'   cards found - so not setting up any additional atom-atom distance restraints
 No 'NOTE BUSTER_UTILANGLE'  cards found - so not setting up any additional restraints on any angle
 No 'NOTE BUSTER_UTILTOR'    cards found - so not setting up any additional sinusoidal restraints on torsions
 
 --- Have some similarity definitions 
     Similarity restraints on RMSD use superposition routines from: 
     Coutsias, E.A., Seok, C., Dill, K.A.(2004)
     "Using quaternions to calculate RMSD", J. Comput. Chem., 25:1849-1857 

 Have found    1 'NOTE BUSTER_SIM_DEFINE' cards 
     Maximum number of chains involved in any SIM group (excluding the template)=     1


 ########## Interpreting card: NOTE BUSTER_SIM_DEFINE ncsautoAB ncsautoABset A
     Number of atoms in template set=  2774
     Number of related chains (other than template) in SIM set=    1
     List of chains to be restrained (excluding template)=   A   
     Pairwise RMSD, XYZ in Angstroms, for SIM group: ncsautoAB
                 B         A   
     B         -----     ----- 
     A         0.580     ----- 
     Overall RMSD,XYZ for the SIM group is     0.580 Angstroms
     Pairwise rms deviation of [individual B's minus their set/chain average] in Angs**2
                 B         A         Bavg
     B         -----     -----     59.76
     A         17.65     -----     74.47
     Overall RMSD,B   for the SIM group is    17.649 Angs**2
     The number of common atoms for the above fits:
                 B         A   
     B         -----     ----- 
     A          2757     ----- 
     (compared to the template set that has    2774 atoms in it)
     List of atoms that are absent in one or more chains for this group: 
                                    B    A   
                    C01     401     +    -
                    N02     401     +    -
                    C03     401     +    -
                    C04     401     +    -
                    N05     401     +    -
                    C06     401     +    -
                    C07     401     +    -
                    C08     401     +    -
                    C09     401     +    -
                    C10     401     +    -
                    C11     401     +    -
                    N12     401     +    -
                    C13     401     +    -
                    O14     401     +    -
                    C15     401     +    -
                    C16     401     +    -
                    C17     401     +    -
     (key  +  atom present for this chain 
           -  atom absent  for this chain)
 transforms for each of the superpositions orthogonal matrix r11 r12 r13 r21 r22 r23 r31 r32 r33 tx ty tz (PDBSET syntax): 
TRANS_BA -0.877967 -0.478604  0.010607 -0.478546  0.878029  0.007656 -0.012978  0.001646 -0.999914    45.374   -12.817    15.707
 reverse transforms 
TRANS_AB -0.877967 -0.478546 -0.012978 -0.478604  0.878029  0.001646  0.010607  0.007656 -0.999914    33.907    32.944    15.322
 
 Setting up LSSR interatomic distance restraints
 Interpreting card: NOTE BUSTER_SIM_RESTRAIN_LSSR ncsautoAB 6.666
        setting up interatomic distance pair restraints with:
        Dmin=         0.00
        Dmax=         5.50
        sigma=        0.20
        Vmax=         3.00
        weight=       6.67
        number of distance pairs defined for this set is      33922
 Have not supplied either '-sim_swap_equiv' or '-sim_swap_equiv_plus' argument 
   so will not try swaping equivalent atoms to improve NCS and/or target agreement.
 
 NCS by LSSR outlier analysis
 -autoncs option automatic NCS prune routine -------- 
 Residues pruned out by gradient condition:
           A|54     A|135     A|145     A|147     A|148     A|149     A|150
          A|151     A|173     A|182     A|187     A|188     A|189     A|215
          A|216     A|217     A|219     A|220     A|234     A|235     A|239
          A|242     A|244     A|246     A|253     A|259     A|260     A|264
          A|288     A|339     A|340     A|344     A|345    A|ENDA      B|54
          B|135     B|145     B|147     B|148     B|149     B|150     B|151
          B|173     B|182     B|187     B|188     B|189     B|215     B|216
          B|217     B|219     B|220     B|234     B|235     B|239     B|242
          B|244     B|246     B|253     B|259     B|260     B|264     B|288
          B|339     B|340     B|344     B|345    B|ENDA
  
 Residues pruned out by function/max condition:
          A|149!    A|187!    A|188!    A|345!   A|ENDA!    B|149!    B|187!
          B|188!    B|345!   B|ENDA!
  
 Total number of residues for which LSSR restraints have been pruned out is       68 out of      698 participating
 Number of LSSR restraints pruned out is      4414 this is   13.0% of the total           33922
  
 -autoncs .Gelly cards suitable for adaptation for manual definition/pruning
           (note auto pruning is done in a slightly different way so exact results may differ slightly)
 -autoncs option .Gelly cards also written to file  auto_gelly_cards.txt
NOTE BUSTER_SET ncsautoXcld = Water    # card added - produced by -autoncs
NOTE BUSTER_SET ncsautoXcld = ncsautoXcld + { B|54 B|135 B|145 B|147 B|148 }    # card added - produced by -autoncs
NOTE BUSTER_SET ncsautoXcld = ncsautoXcld + { B|149 B|150 B|151 B|173 B|182 }    # card added - produced by -autoncs
NOTE BUSTER_SET ncsautoXcld = ncsautoXcld + { B|187 B|188 B|189 B|215 B|216 }    # card added - produced by -autoncs
NOTE BUSTER_SET ncsautoXcld = ncsautoXcld + { B|217 B|219 B|220 B|234 B|235 }    # card added - produced by -autoncs
NOTE BUSTER_SET ncsautoXcld = ncsautoXcld + { B|239 B|242 B|244 B|246 B|253 }    # card added - produced by -autoncs
NOTE BUSTER_SET ncsautoXcld = ncsautoXcld + { B|259 B|260 B|264 B|288 B|339 }    # card added - produced by -autoncs
NOTE BUSTER_SET ncsautoXcld = ncsautoXcld + { B|340 B|344 B|345 B|ENDA }    # card added - produced by -autoncs
NOTE BUSTER_SET ncsautoABset = Chain_B \ ncsautoXcld    # card added - produced by -autoncs
NOTE BUSTER_SIM_DEFINE ncsautoAB ncsautoABset A    # card added - produced by -autoncs
NOTE BUSTER_SIM_RESTRAIN_LSSR ncsautoAB 6.666    # card added - produced by -autoncs

 LSSR function contribution before pruning     26397.5
                             after pruning      9763.0
 Graph of which residues pruned from LSSR: ncs_pruned.mtv
 Graph of LSSR function outliers ncs_function_outliers.mtv
 Graph of LSSR gradient outliers ncs_gradient_outliers.mtv
 LSSR - analysis of distance difference distribution for NCS
         DeltaD less than 0.5 Angs:  proportion of population= 98.67% average DeltaD of subset     0.008 Angs
 Histogram of LSSR deltaD distribution ncs_histogram.mtv
 
 Interpreting card: NOTE BUSTER_TLS_SET tls1 { A|* }
 Interpreting card: NOTE BUSTER_TLS_SET tls2 { B|* }
 Interpreting card: NOTE BUSTER_TLS_SET tls1 { A|* }
 Interpreting card: NOTE BUSTER_TLS_T   tls1     -2.09805E-01 -1.15711E-01  3.73362E-01 -2.40920E-02  4.96139E-02  2.21940E-02
 Interpreting card: NOTE BUSTER_TLS_L   tls1      1.24514E+00  1.05226E+00  6.66191E-01  1.62361E-01 -3.88521E-01  3.54587E-01
 Interpreting card: NOTE BUSTER_TLS_S   tls1      2.06506E-01 -1.53727E-01  1.93700E-01 -1.05534E-01 -6.67227E-02 -9.37109E-02 -4.76199E-02 -4.26923E-02
 Interpreting card: NOTE BUSTER_TLS_O   tls1     -2.33748E+01 -8.36624E+01  1.14308E+01
 Interpreting card: NOTE BUSTER_TLS_SET tls2 { B|* }
 Interpreting card: NOTE BUSTER_TLS_T   tls2     -2.19975E-01 -1.58351E-01  4.94077E-01  1.93068E-02  4.62273E-02  2.44113E-03
 Interpreting card: NOTE BUSTER_TLS_L   tls2      1.30038E+00  8.42752E-01  9.70856E-01  6.65317E-01 -8.38129E-01 -5.84650E-01
 Interpreting card: NOTE BUSTER_TLS_S   tls2     -2.69202E-02  1.09150E-01 -8.14403E-02  6.77598E-02  1.92600E-01  1.97964E-02 -6.29584E-02  2.14661E-02
 Interpreting card: NOTE BUSTER_TLS_O   tls2      2.20570E+01 -7.19562E+01  3.49479E+00
 Interpreting card: NOTE BUSTER_TLS_FIX tls1 ALL
 Interpreting card: NOTE BUSTER_TLS_FIX tls2 ALL


 TLS information. Total number of groups:     2


 Group   Natoms    FixRB   FixTLS   Group Tag    Group Spec
     1     2770      Y        Y     tls1    { A|* }
     2     2774      Y        Y     tls2    { B|* }
 
NOTE BUSTER_TLS_SET tls1 { A|* }
NOTE BUSTER_TLS_T   tls1     -2.09805E-01 -1.15711E-01  3.73362E-01 -2.40920E-02  4.96139E-02  2.21940E-02
NOTE BUSTER_TLS_L   tls1      1.24514E+00  1.05226E+00  6.66191E-01  1.62361E-01 -3.88521E-01  3.54587E-01
NOTE BUSTER_TLS_S   tls1      2.06506E-01 -1.53727E-01  1.93700E-01 -1.05534E-01 -6.67227E-02 -9.37109E-02 -4.76199E-02 -4.26923E-02
NOTE BUSTER_TLS_O   tls1     -2.33748E+01 -8.36624E+01  1.14308E+01
NOTE BUSTER_TLS_FIX tls1 ALL
 
NOTE BUSTER_TLS_SET tls2 { B|* }
NOTE BUSTER_TLS_T   tls2     -2.19975E-01 -1.58351E-01  4.94077E-01  1.93068E-02  4.62273E-02  2.44113E-03
NOTE BUSTER_TLS_L   tls2      1.30038E+00  8.42752E-01  9.70856E-01  6.65317E-01 -8.38129E-01 -5.84650E-01
NOTE BUSTER_TLS_S   tls2     -2.69202E-02  1.09150E-01 -8.14403E-02  6.77598E-02  1.92600E-01  1.97964E-02 -6.29584E-02  2.14661E-02
NOTE BUSTER_TLS_O   tls2      2.20570E+01 -7.19562E+01  3.49479E+00
NOTE BUSTER_TLS_FIX tls2 ALL


 TLS: mmCIF group specification begin


#
loop_
_pdbx_refine_tls_group.id
_pdbx_refine_tls_group.refine_tls_id
_pdbx_refine_tls_group.beg_PDB_ins_code
_pdbx_refine_tls_group.beg_auth_asym_id
_pdbx_refine_tls_group.beg_auth_seq_id
_pdbx_refine_tls_group.end_PDB_ins_code
_pdbx_refine_tls_group.end_auth_asym_id
_pdbx_refine_tls_group.end_auth_seq_id
_pdbx_refine_tls_group.selection
_pdbx_refine_tls_group.selection_details
   1   1 . A   -1 . A  346 . '{ A|* }'
   2   1 . A  401 . A  401 . '{ A|* }'
   3   2 . B   -1 . B  346 . '{ B|* }'
   4   2 . B  401 . B  401 . '{ B|* }'
#


 TLS: mmCIF group specification end


 Interpreting card: NOTE BUSTER_ANISO_SET Empty
 Interpreting card: NOTE BUSTER_ANISO_WEIGHT SCA  16.0
 Interpreting card: NOTE BUSTER_ANISO_WEIGHT SPH   0.6    0.6
 Interpreting card: NOTE BUSTER_ANISO_WEIGHT SIM   7.8    4.8    2.6    0
 Interpreting card: NOTE BUSTER_ANISO_WEIGHT RIG  21.5   21.5    3.7    0


 After TLS and ANISO: Number of atoms with fixed/combined xyz =     0
 After TLS and ANISO: Number of atoms with fixed/combined B   =     0
 After TLS and ANISO: Number of atoms with fixed/combined OCC =  6125
 After TLS and ANISO: Number of atoms with fixed/combined BIJ =  6125
 After TLS and ANISO: Number of atoms in a TLS group          =  5544
                                     cf total number of atoms =  6125
 

 ############### Setup state vector

 No free_vect_ulim_xyz argument found: using default of  100.000
 No free_vect_llim_xyz argument found: using default of -100.000
 Number of parameters added for TLS description                 =       0
 Number of parameters to describe molecular conformation        =   24500

 ############### Setup free vector

 Number of free parameters to be adjusted in refinement   =   24500

 ############### Setting up own geometry


 No command line argument '-tnt_fn' so will use gelly to do geometry function calculations

 ============= Call TNT functions


 ============= Get TNT symmetry information

 
      Getting symmetry operators from TNT. 
          gelly will classify symmetry using pdb-like convention: 
               SYMOP   SYMMETRY
              NNNMMM   OPERATOR
                1555   X,Y,Z
                2555   -Y,X-Y,Z
                3555   -X+Y,-X,Z
                4555   -X,-Y,1/2+Z
                5555   Y,-X+Y,1/2+Z
                6555   X-Y,X,1/2+Z
          where NNN -> operator number and MMM -> translation vector
 

 ============= Own Gelly geometry/restraints setup


 ----------- Identify bond-types


    Setting up GEOMBONDREC_TYPE_HASH* to be able to map a given bond type (e.g.
    "single") to an internal integer (GBOND_TYPE=1) etc.

    Using default  GEOMBONDREC_TYPE_HASH_STR='SING* 1 DOUB* 2 TRIP* 3 AROM* 4 DELO* 4 METAL* 5':
    to override default use a NOTE BUSTER_GEOMBONDREC_TYPE_HASH_STR gelly card.

 ----------- Classify torsion into peptide omega and other


 ----------- Setup of non-bonded terms

 Ideal-distance contact term setup:
    Restraint function constants (normally these values are from NOTE BUSTER_IDEAL_CONTACT_* cards)
    Sigma for general contacts      0.200 Angs
    Sigma for 1-4 contacts          0.100 Angs (contacts across a torsion)
    Sigma for 1-5 contacts          0.150 Angs
    Sigma for 1-N contacts          0.200 Angs
    Sigma for HBOND pairs           0.100 Angs (contacts between HB_types A and D or B's)
    Contact distance increment for each atom in HBOND          -0.150 Angs 
    Contact distance increment for H-bond acceptor              0.100 Angs 
    Contact distance increment for 1-4 with an O atom          -0.050 Angs 
    Contact distance increment for 1-4 with an N atom          -0.180 Angs 
    Contact distance increment for 1-4 with an C atom          -0.370 Angs 
    Contact distance increment for 1-4 with an S atom          -0.150 Angs 
    Contact distance increment for 1-4 with an P atom          -0.150 Angs 
    Contact distance increment for 1-4 with any other atom     -0.150 Angs 
    All atoms matched for CCP4 atom type.
    Number of CCP4 atom types used is         24 (H-bound and not H-bound counted separately)

 Setting up short/ideal-distance contacts
   Using gelly special position handling. (For TNT instead rerun with argument '-special_tnt')

   No command line argument '-special_all' specified. We will auto-generate
   EXCLUDE cards only for atom-atom contacts across symmetry (i.e. on special
   position) when this involves a contact within a residue to itself.

   Have set up non-bonded exclusions - 1-3 contacts
   Atom A|27:CA         makes the most 1-3 contacts:  6

   Have set up non-bonded exclusions - 1-4 contacts
   Atom A|27:N          makes the most 1-4 contacts:  7

   Have set up non-bonded exclusions - 1-5 contacts
   Atom A|28:C.A        makes the most 1-5 contacts: 10

 ############################################################################################################
 ###################################################### setting up contact terms
 ############################################################################################################


 ============= Call Gelly versions of TNT functions

 WARNING: 1-4 contact without 1-(2,3)-4 data for A|401:O2(CIT)        and A|401:C3(CIT)        X-X
 WARNING: 1-4 contact without 1-(2,3)-4 data for A|401:C2(CIT)        and A|401:O6(CIT)        X-X
 WARNING: 1-4 contact without 1-(2,3)-4 data for A|401:O7(CIT)        and A|401:O5(CIT)        X-X
 WARNING: 1-4 contact without 1-(2,3)-4 data for A|401:C4(CIT)        and A|401:O6(CIT)        X-X
 WARNING: 1-4 contact without 1-(2,3)-4 data for B|401:C03(LIG)       and B|401:C06(LIG)       X-X
 WARNING: 1-4 contact without 1-(2,3)-4 data for B|401:C04(LIG)       and B|401:C07(LIG)       X-X

       TERM      weight      number      TNT_fn    Gelly_fn        diff
       BOND       2.000        5660     663.417     663.417       0.000
       ANGL       2.000        7681    3850.843    3850.843       0.000
       IMPR       0.000           0       0.000       0.000       0.000
       TORS       2.000        1968    2968.908    2968.890       0.018
       TRIG       2.000           0       0.000       0.000       0.000
       PLAN       5.000         964    1525.020    1525.020       0.000
       CONT       5.000           0     122.145       0.000     122.145
       BCOR      10.000        5630    1345.268    3179.868   -1834.600


 ============= Call Gelly geometry functions

   Switching torsion functional form to sinusoidal rather than split harmonic functional.
      if you want this not to happen then rerun with '-torsharm'
      Weight for sinusoidal torsion=     2.000
   BCOR function/gradient will be split between H and non-H atoms (as for any atom pair). Use '-bcorrel_all_in_hydrogens' to change this.

 ----------- Fix B-correlation restraints

 
 Checking for bonds that do not have a bcorrel restraint defined
 We will wipe all existing BCORrels if OCC=0.0 atom(s) is/are involved. This can be' switched off via the '-bcorrel_nowipe_zeroocc' flag.
 We will wipe all existing BCORrels if only non-hydrogen atom(s) with fixed B(iso) are involved. This can be switched off via the '-bcorrel_nowipe_fixb_nonhydrogens' flag.
 We will keep all existing BCORrels if hydrogen atom(s) with fixed B(iso) is/are involved. This can be switched off via the '-bcorrel_wipe_fixb_hydrogens' flag.
 No -bcorrel_missing_sigma found. So will set sigma to     2.5 Ang**2 for any bond with no existing BCORrel
 No -bcorrel_missing_sigma_hydrogens found. So will set sigma to     1.0 Ang**2 for any bond with no existing BCORrel involving hydrogens

 No active BCORels found involving OCC=0.0 atom(s)


 No active BCORels found involving fixed-B (only) non-hydrogen atom(s)

    add a default BCOR for bond A|401:C1(CIT) A|401:C2(CIT)
    add a default BCOR for bond A|401:C1(CIT) A|401:O1(CIT)
    add a default BCOR for bond A|401:C1(CIT) A|401:O2(CIT)
    add a default BCOR for bond A|401:C2(CIT) A|401:C3(CIT)
    add a default BCOR for bond A|401:C3(CIT) A|401:C4(CIT)
    add a default BCOR for bond A|401:C3(CIT) A|401:C6(CIT)
    add a default BCOR for bond A|401:C3(CIT) A|401:O7(CIT)
    add a default BCOR for bond A|401:C4(CIT) A|401:C5(CIT)
    add a default BCOR for bond A|401:C5(CIT) A|401:O3(CIT)
    add a default BCOR for bond A|401:C5(CIT) A|401:O4(CIT)
    add a default BCOR for bond A|401:C6(CIT) A|401:O5(CIT)
    add a default BCOR for bond A|401:C6(CIT) A|401:O6(CIT)
    add a default BCOR for bond B|401:C01(LIG) B|401:N02(LIG)
    add a default BCOR for bond B|401:N02(LIG) B|401:C03(LIG)
    add a default BCOR for bond B|401:N02(LIG) B|401:C07(LIG)
    add a default BCOR for bond B|401:C03(LIG) B|401:C04(LIG)
    add a default BCOR for bond B|401:C04(LIG) B|401:N05(LIG)
    add a default BCOR for bond B|401:N05(LIG) B|401:C06(LIG)
    add a default BCOR for bond B|401:N05(LIG) B|401:C08(LIG)
    add a default BCOR for bond B|401:C06(LIG) B|401:C07(LIG)
    add a default BCOR for bond B|401:C08(LIG) B|401:C09(LIG)
    add a default BCOR for bond B|401:C08(LIG) B|401:C17(LIG)
    add a default BCOR for bond B|401:C09(LIG) B|401:C10(LIG)
    add a default BCOR for bond B|401:C10(LIG) B|401:C11(LIG)
    add a default BCOR for bond B|401:C11(LIG) B|401:N12(LIG)
    add a default BCOR for bond B|401:C11(LIG) B|401:C16(LIG)
    add a default BCOR for bond B|401:N12(LIG) B|401:C13(LIG)
    add a default BCOR for bond B|401:C13(LIG) B|401:O14(LIG)
    add a default BCOR for bond B|401:C13(LIG) B|401:C15(LIG)
    add a default BCOR for bond B|401:C16(LIG) B|401:C17(LIG)

 Have added a total of        30 BCORrels for bonds which do not already have a BCORrel


 No bonds (for potential BCORrel addition) were skipped due to both atoms having constant/fixed B(iso)


 ----------- Deactivate hydrogen-free planes if hydrogen atoms present

 A total of       0 planes have been deactivated because they are duplicated hydrogen-free and with-hydrogen forms.

 ----------- Handle peptide omega angle(s)

      Torsion angles split into peptide omega and other
         Number of peptide omega angles is       698
         Number of other torsion is             1270
      Peptide omega angles are monitored rather than directly restrained (PLANE used instead).

 ----------- Fix cis-PRO

 Now apply the fix to get correct restraints for cis PRO residues
   Have got      11 bonds records and      26 angles
   from GEOMETRY cards for the special PROC residue
   Weight for ideal-distance contact term=     4.000

 ----------- Prepare SSBOND/LINK for output PDB file


 ----------- Prepare for QM


 ----------- Prepare symmetry in utility distance restraints


 ############### Setting up Rhofit contacts (if needed)

 No ligand-(fixed protein) contact term will be used

 ############### Working out initial function and gradient


   MaxLik Scaling Cycle 0002
 
 Initial functional value = 5.522313E+05 rms gradient=      57.6817
 X-ray contribution to function value = 5.235194E+05
 Geometry contribution (inc ncs)      =      28711.9
 Geometry function summary
     term                           number     weight       rms        GooF      function     fn/numb
     ================================================================================================
     BOND bond lengths (angs)       5660       2.000      0.0049       0.242       663.42       0.117
     ANGL bond angles (degs)        7681       2.000        0.84       0.501      3850.84       0.501
     TORS split-harmonic tors       1270       0.000       17.19       1.081         0.00       0.000
     SINTOR sinusoidal tors         1270       2.000       17.19                  2288.82       1.802
     PLAN general plane (angs)       964       5.000      0.0112       0.562      1525.02       1.582
     BCORrelations (angs^2)         5660      10.000       0.665       0.237      3187.10       0.563
     CONT (bad contacts)               0       5.000                                 0.00
     IDEAL(ideal-dist contact)      4882       4.000                              7403.29
     CHIRAL (gelly semiharmon)       740       5.000                                30.41
     SIM similarity restraint for NCS and/or TARGET                               9763.00
     OCCSUM restraint on sum of occupanies                                           0.00       as none defined
     DISTAN utility restraints interatomic distance                                  0.00       as none defined
     UTILANGLE utility angle restraints                                              0.00       as none defined
     UTILTOR utility restraints on torsion angles                                    0.00       as none defined
     Peptide omega torsion angles                           2.39 degs - monitor only
 
 WARNING: Have |delta/sigma| deviations > 5.0 sigma. Number of outliers for each term: 
 WARNING:         2 bond angles.  Worst is     5.3 sigs   135.03 degs A|28:C.A(ASN)=A|29:N(ASN)=A|29:CA(ASN)

 Weighted rms      0.0049         0.840  (temporary rms bond and angle for BUSTER to pick up!)
 Weighted intra-residual rms(bond) for StdProtein (excluding H)      0.0047
 Weighted intra-residual rms(bond) for Buffer     (excluding H)      0.0029

 ############### Running initial check


 Initial Geometry Sanity Check

 Check for duplicate bonds: no duplicates found
 
 Check for duplicate or missing bond angles: all are OK
 Check for bond between an atom and itself not involving symmetry:  all are OK
   There are no bad contacts where Delta/sigma is greater than     6.5
   Worst contact dist=   2.819 std=   3.185 sigma=   0.107 abs(Delta/sigma)=    3.419 ats B|28:CB.B (ASN) B|29:N (ASN)
   It is likely that the geometry libraries, sequence info and links match the input molecule
 GEOMETRY CHECK OVERALL:  PASS


 ############### Creating geometry/screen report


 ############### Option "tao" - run Tao optimizer

 TAO: Toolkit for Advanced Optimization. Reference: 
     Munson, T., Sarich, J., Wild, S., Benson, S., McInnes, L.C. (2018)
     "TAO User Manual (Revision 3.9)", Mathematics and Computer Science
     Division, Argonne National Laboratory
     ANL/MCS-TM-322, http://www.mcs.anl.gov/petsc/
 
 Have found -keeppdb flag so will write out a .pdb file every 1000000 its during opt
 Will apply TAO optimization 
 Maximum number of iterations      100
 Convergence test on Grms       4.0000 (-glim)
 Limit on rms displacement to original position (rmsD2init): NONE test inactive (-dlim)

    analysing argument no 0 : -autoncs with j=0
    analysing argument no 1 : -hydrogens_input_occ2zero with j=0
    analysing argument no 2 : -hydrogens_output_occ2heavy with j=0
    analysing argument no 3 : -screen with j=0
    analysing argument no 4 : 100 with j=0
    analysing argument no 5 : -screen_sigma with j=0
    analysing argument no 6 : 3.0 with j=0
    analysing argument no 7 : -keeppdb with j=0
    analysing argument no 8 : 1000000 with j=0
    analysing argument no 9 : -taomonitor_verbose with j=0
    found argument starting with "-tao" : -taomonitor_verbose
    analysing argument no 10 : 2 with j=1
    found value after -tao/-options_/-viewer_ argument : "2"
 PETSC/TAO: setting option "-taomonitor_verbose" = "2"


   MaxLik Scaling Cycle 0003
   Iters     Tot_Funct    Xray_Funct    Geom_Funct          Grms     rmsD2init     maxD2init             R         Rfree           LLG       LLGfree     rms(bond)    rms(angle)       Opttag_H
   Iters             K   B_IMPF_FRAG        K_MISS   B_TEMP_MISS   K_IMPF_MISS   B_IMPF_MISS        K_SOLV   B_TEMP_SOLV   K_IMPF_SOLV   B_IMPF_SOLV        B_TEMP     B_TEMP_11     B_TEMP_12     B_TEMP_22     B_TEMP_13     B_TEMP_23     B_TEMP_33      K_WILSON      B_WILSON      FGCYCLES       Scatag_H
   Iters          BOND         ANGLE      IMPROPER       TORSION    SINTORSION      TRIGONAL        PLANAR  BCORRELATION       CONTACT     IDEALDIST        CHIRAL    SIMILARITY       OCCSSUM  UTILDISTANCE     UTILANGLE   UTILTORSION        RHOFIT        ADP-01        ADP-02        ADP-03        ADP-04        ADP-05        ADP-06        ADP-07        ADP-08        ADP-09        ADP-10      RHOFITPC            QM       Funtag_H
       0  5.522313E+05  5.235194E+05       28711.9       57.6817  0.000000E+00  0.000000E+00      0.212901      0.229248  0.000000E+00  0.000000E+00    0.00485774      0.840427       Opttag_D
       0       56.7116       3.29402       1.00000  0.000000E+00       1.00000  0.000000E+00      0.898189       59.3818       1.00000       16.5265  0.000000E+00       2.27926  0.000000E+00       2.27926  0.000000E+00  0.000000E+00      -4.55852       56.3253       2.16394        2       Scatag_D
       0       663.417       3850.84  0.000000E+00  0.000000E+00       2288.82  0.000000E+00       1525.02       3187.10  0.000000E+00       7403.29       30.4090       9763.00 -1.000000E+10 -1.000000E+10 -1.000000E+10 -1.000000E+10 -1.000000E+10  0.000000E+00  0.000000E+00  0.000000E+00  0.000000E+00  0.000000E+00  0.000000E+00  0.000000E+00  0.000000E+00  0.000000E+00  0.000000E+00  0.000000E+00  0.000000E+00       Funtag_D
   Geometry Restraint Deviations SCREEN Information

 

 An atom has moved more than     1.50 A from the initial/last
 position - if this was an accepted trial point, probably need to update the
 non-bonded contact list.


   MaxLik Scaling Cycle 0004

   MaxLik Scaling Cycle 0005

   MaxLik Scaling Cycle 0006

   Wilson plot

   Statistics in resolution bins after refinement cycle 0001
```


|  |  |
| --- | --- |
|  | - Rxpct factors plot- Rxpct factors table- <Log-Lik Gain>- Ln Sigma A- Correlation Coefficients |


```
   Iters     Tot_Funct    Xray_Funct    Geom_Funct          Grms     rmsD2init     maxD2init             R         Rfree           LLG       LLGfree     rms(bond)    rms(angle)
   Iters             K   B_IMPF_FRAG        K_MISS   B_TEMP_MISS   K_IMPF_MISS   B_IMPF_MISS        K_SOLV   B_TEMP_SOLV   K_IMPF_SOLV   B_IMPF_SOLV        B_TEMP     B_TEMP_11     B_TEMP_12     B_TEMP_22     B_TEMP_13     B_TEMP_23     B_TEMP_33      K_WILSON      B_WILSON      FGCYCLES
   Iters          BOND         ANGLE      IMPROPER       TORSION    SINTORSION      TRIGONAL        PLANAR  BCORRELATION       CONTACT     IDEALDIST        CHIRAL    SIMILARITY       OCCSSUM  UTILDISTANCE     UTILANGLE   UTILTORSION        RHOFIT        ADP-01        ADP-02        ADP-03        ADP-04        ADP-05        ADP-06        ADP-07        ADP-08        ADP-09        ADP-10      RHOFITPC            QM
       1  5.514748E+05  5.226478E+05       28826.9       53.8903  9.448504E-04    0.00395684      0.212379      0.229115    0.00219767  6.073779E-04    0.00505219      0.841704       Opttag_D
       1       56.6709       3.23788       1.00000  0.000000E+00       1.00000  0.000000E+00      0.897959       59.1139       1.00000       16.6216  0.000000E+00       2.27662  0.000000E+00       2.27662  0.000000E+00  0.000000E+00      -4.55324       56.3253       2.16394        3       Scatag_D
       1       717.591       3862.55  0.000000E+00  0.000000E+00       2288.25  0.000000E+00       1540.51       3203.66  0.000000E+00       7410.47       31.7565       9772.11 -1.000000E+10 -1.000000E+10 -1.000000E+10 -1.000000E+10 -1.000000E+10  0.000000E+00  0.000000E+00  0.000000E+00  0.000000E+00  0.000000E+00  0.000000E+00  0.000000E+00  0.000000E+00  0.000000E+00  0.000000E+00  0.000000E+00  0.000000E+00       Funtag_D
   Geometry Restraint Deviations SCREEN Information

 

   MaxLik Scaling Cycle 0007

   Wilson plot

   Statistics in resolution bins after refinement cycle 0002
```


|  |  |
| --- | --- |
|  | - Rxpct factors plot- Rxpct factors table- <Log-Lik Gain>- Ln Sigma A- Correlation Coefficients |


```
   Iters     Tot_Funct    Xray_Funct    Geom_Funct          Grms     rmsD2init     maxD2init             R         Rfree           LLG       LLGfree     rms(bond)    rms(angle)
   Iters             K   B_IMPF_FRAG        K_MISS   B_TEMP_MISS   K_IMPF_MISS   B_IMPF_MISS        K_SOLV   B_TEMP_SOLV   K_IMPF_SOLV   B_IMPF_SOLV        B_TEMP     B_TEMP_11     B_TEMP_12     B_TEMP_22     B_TEMP_13     B_TEMP_23     B_TEMP_33      K_WILSON      B_WILSON      FGCYCLES
   Iters          BOND         ANGLE      IMPROPER       TORSION    SINTORSION      TRIGONAL        PLANAR  BCORRELATION       CONTACT     IDEALDIST        CHIRAL    SIMILARITY       OCCSSUM  UTILDISTANCE     UTILANGLE   UTILTORSION        RHOFIT        ADP-01        ADP-02        ADP-03        ADP-04        ADP-05        ADP-06        ADP-07        ADP-08        ADP-09        ADP-10      RHOFITPC            QM
       2  5.508799E+05  5.218659E+05       29014.1       43.3959    0.00202820    0.00908170      0.211860      0.229010    0.00416932    0.00111358    0.00533972      0.843909       Opttag_D
       2       56.6314       3.18949       1.00000  0.000000E+00       1.00000  0.000000E+00      0.897756       58.9484       1.00000       16.6593  0.000000E+00       2.26804  0.000000E+00       2.26804  0.000000E+00  0.000000E+00      -4.53608       56.3253       2.16394        1       Scatag_D
       2       801.596       3882.82  0.000000E+00  0.000000E+00       2287.53  0.000000E+00       1563.13       3228.14  0.000000E+00       7425.21       32.7938       9792.86 -1.000000E+10 -1.000000E+10 -1.000000E+10 -1.000000E+10 -1.000000E+10  0.000000E+00  0.000000E+00  0.000000E+00  0.000000E+00  0.000000E+00  0.000000E+00  0.000000E+00  0.000000E+00  0.000000E+00  0.000000E+00  0.000000E+00  0.000000E+00       Funtag_D
   Geometry Restraint Deviations SCREEN Information

 
       3  5.503175E+05  5.207155E+05       29602.0       43.1822    0.00380787     0.0177503      0.211077      0.228863    0.00706986    0.00183599    0.00582543      0.848947       Opttag_D
       3       56.5718       3.12058       1.00000  0.000000E+00       1.00000  0.000000E+00      0.897455       58.7558       1.00000       16.6879  0.000000E+00       2.25403  0.000000E+00       2.25403  0.000000E+00  0.000000E+00      -4.50807       56.3253       2.16394        1       Scatag_D
       3       954.059       3929.32  0.000000E+00  0.000000E+00       2286.48  0.000000E+00       1606.25       3492.27  0.000000E+00       7456.78       33.6411       9843.18 -1.000000E+10 -1.000000E+10 -1.000000E+10 -1.000000E+10 -1.000000E+10  0.000000E+00  0.000000E+00  0.000000E+00  0.000000E+00  0.000000E+00  0.000000E+00  0.000000E+00  0.000000E+00  0.000000E+00  0.000000E+00  0.000000E+00  0.000000E+00       Funtag_D
       4  5.499142E+05  5.191949E+05       30719.3       65.7628    0.00639902     0.0311704      0.210029      0.228653     0.0109039    0.00277807    0.00646548      0.867206       Opttag_D
       4       56.4916       3.03267       1.00000  0.000000E+00       1.00000  0.000000E+00      0.897059       58.5501       1.00000       16.6987  0.000000E+00       2.23626  0.000000E+00       2.23626  0.000000E+00  0.000000E+00      -4.47253       56.3253       2.16394        1       Scatag_D
       4       1175.22       4100.15  0.000000E+00  0.000000E+00       2285.27  0.000000E+00       1679.10       3988.90  0.000000E+00       7518.34       33.8094       9938.52 -1.000000E+10 -1.000000E+10 -1.000000E+10 -1.000000E+10 -1.000000E+10  0.000000E+00  0.000000E+00  0.000000E+00  0.000000E+00  0.000000E+00  0.000000E+00  0.000000E+00  0.000000E+00  0.000000E+00  0.000000E+00  0.000000E+00  0.000000E+00       Funtag_D
       5  5.494867E+05  5.186345E+05       30852.3       38.8948    0.00750201     0.0374561      0.209629      0.228541     0.0123170    0.00319047    0.00654528      0.859772       Opttag_D
       5       56.4598       3.00132       1.00000  0.000000E+00       1.00000  0.000000E+00      0.896908       58.5056       1.00000       16.6856  0.000000E+00       2.22998  0.000000E+00       2.22998  0.000000E+00  0.000000E+00      -4.45996       56.3253       2.16394        1       Scatag_D
       5       1204.41       4030.16  0.000000E+00  0.000000E+00       2284.72  0.000000E+00       1713.50       4064.84  0.000000E+00       7548.73       33.2987       9972.59 -1.000000E+10 -1.000000E+10 -1.000000E+10 -1.000000E+10 -1.000000E+10  0.000000E+00  0.000000E+00  0.000000E+00  0.000000E+00  0.000000E+00  0.000000E+00  0.000000E+00  0.000000E+00  0.000000E+00  0.000000E+00  0.000000E+00  0.000000E+00       Funtag_D
       6  5.490683E+05  5.179355E+05       31132.8       39.7916    0.00912469     0.0468420      0.209117      0.228376     0.0140794    0.00377225    0.00659386      0.866022       Opttag_D
       6       56.4179       2.96312       1.00000  0.000000E+00       1.00000  0.000000E+00      0.896704       58.4840       1.00000       16.6527  0.000000E+00       2.22171  0.000000E+00       2.22171  0.000000E+00  0.000000E+00      -4.44341       56.3253       2.16394        1       Scatag_D
       6       1222.36       4088.97  0.000000E+00  0.000000E+00       2284.05  0.000000E+00       1764.15       4133.30  0.000000E+00       7597.18       32.5195       10010.3 -1.000000E+10 -1.000000E+10 -1.000000E+10 -1.000000E+10 -1.000000E+10  0.000000E+00  0.000000E+00  0.000000E+00  0.000000E+00  0.000000E+00  0.000000E+00  0.000000E+00  0.000000E+00  0.000000E+00  0.000000E+00  0.000000E+00  0.000000E+00       Funtag_D
       7  5.486899E+05  5.169941E+05       31695.9       54.0143     0.0115367     0.0605324      0.208424      0.228134     0.0164531    0.00458108    0.00670783      0.879017       Opttag_D
       7       56.3625       2.91279       1.00000  0.000000E+00       1.00000  0.000000E+00      0.896417       58.4737       1.00000       16.6025  0.000000E+00       2.20832  0.000000E+00       2.20832  0.000000E+00  0.000000E+00      -4.41664       56.3253       2.16394        1       Scatag_D
       7       1264.98       4212.60  0.000000E+00  0.000000E+00       2283.19  0.000000E+00       1838.89       4328.42  0.000000E+00       7672.87       32.4555       10062.4 -1.000000E+10 -1.000000E+10 -1.000000E+10 -1.000000E+10 -1.000000E+10  0.000000E+00  0.000000E+00  0.000000E+00  0.000000E+00  0.000000E+00  0.000000E+00  0.000000E+00  0.000000E+00  0.000000E+00  0.000000E+00  0.000000E+00  0.000000E+00       Funtag_D
       8  5.482597E+05  5.157564E+05       32503.2       47.2892     0.0149692     0.0796065      0.207529      0.227832     0.0195737    0.00559995    0.00686509      0.887416       Opttag_D
       8       56.2920       2.84815       1.00000  0.000000E+00       1.00000  0.000000E+00      0.896021       58.4752       1.00000       16.5363  0.000000E+00       2.18898  0.000000E+00       2.18898  0.000000E+00  0.000000E+00      -4.37796       56.3253       2.16394        1       Scatag_D
       8       1324.98       4293.49  0.000000E+00  0.000000E+00       2282.00  0.000000E+00       1946.52       4702.27  0.000000E+00       7786.94       33.3968       10133.6 -1.000000E+10 -1.000000E+10 -1.000000E+10 -1.000000E+10 -1.000000E+10  0.000000E+00  0.000000E+00  0.000000E+00  0.000000E+00  0.000000E+00  0.000000E+00  0.000000E+00  0.000000E+00  0.000000E+00  0.000000E+00  0.000000E+00  0.000000E+00       Funtag_D
       9  5.478833E+05  5.150934E+05       32789.9       39.1441     0.0170416     0.0910741      0.207053      0.227651     0.0212454    0.00620101    0.00690399      0.891252       Opttag_D
       9       56.2553       2.81401       1.00000  0.000000E+00       1.00000  0.000000E+00      0.895758       58.4911       1.00000       16.5053  0.000000E+00       2.17834  0.000000E+00       2.17834  0.000000E+00  0.000000E+00      -4.35668       56.3253       2.16394        1       Scatag_D
       9       1340.04       4330.69  0.000000E+00  0.000000E+00       2281.32  0.000000E+00       2003.52       4789.54  0.000000E+00       7856.52       34.8254       10153.5 -1.000000E+10 -1.000000E+10 -1.000000E+10 -1.000000E+10 -1.000000E+10  0.000000E+00  0.000000E+00  0.000000E+00  0.000000E+00  0.000000E+00  0.000000E+00  0.000000E+00  0.000000E+00  0.000000E+00  0.000000E+00  0.000000E+00  0.000000E+00       Funtag_D
      10  5.474250E+05  5.142095E+05       33215.5       39.4450     0.0200698      0.107425      0.206435      0.227388     0.0234741    0.00702339    0.00695392      0.899068       Opttag_D
      10       56.2076       2.76888       1.00000  0.000000E+00       1.00000  0.000000E+00      0.895330       58.5267       1.00000       16.4718  0.000000E+00       2.16469  0.000000E+00       2.16469  0.000000E+00  0.000000E+00      -4.32938       56.3253       2.16394        1       Scatag_D
      10       1359.50       4406.98  0.000000E+00  0.000000E+00       2280.47  0.000000E+00       2077.15       4927.94  0.000000E+00       7958.18       37.7067       10167.5 -1.000000E+10 -1.000000E+10 -1.000000E+10 -1.000000E+10 -1.000000E+10  0.000000E+00  0.000000E+00  0.000000E+00  0.000000E+00  0.000000E+00  0.000000E+00  0.000000E+00  0.000000E+00  0.000000E+00  0.000000E+00  0.000000E+00  0.000000E+00       Funtag_D
      11  5.470258E+05  5.133188E+05       33706.9       39.3734     0.0234781      0.125256      0.205820      0.227120     0.0257199    0.00784201    0.00700489      0.907468       Opttag_D
      11       56.1596       2.72387       1.00000  0.000000E+00       1.00000  0.000000E+00      0.894733       58.5950       1.00000       16.4399  0.000000E+00       2.15249  0.000000E+00       2.15249  0.000000E+00  0.000000E+00      -4.30498       56.3253       2.16394        1       Scatag_D
      11       1379.50       4489.71  0.000000E+00  0.000000E+00       2279.46  0.000000E+00       2150.11       5114.59  0.000000E+00       8073.07       40.0852       10180.4 -1.000000E+10 -1.000000E+10 -1.000000E+10 -1.000000E+10 -1.000000E+10  0.000000E+00  0.000000E+00  0.000000E+00  0.000000E+00  0.000000E+00  0.000000E+00  0.000000E+00  0.000000E+00  0.000000E+00  0.000000E+00  0.000000E+00  0.000000E+00       Funtag_D
      12  5.465766E+05  5.123387E+05       34237.9       41.5088     0.0277844      0.146980      0.205247      0.226675     0.0281911    0.00890635    0.00705500      0.919005       Opttag_D
      12       56.0743       2.67633       1.00000  0.000000E+00       1.00000  0.000000E+00      0.879999       60.3695       1.00000       16.3173  0.000000E+00       2.13741  0.000000E+00       2.13741  0.000000E+00  0.000000E+00      -4.27483       56.3253       2.16394        1       Scatag_D
      12       1399.31       4604.60  0.000000E+00  0.000000E+00       2278.85  0.000000E+00       2223.33       5283.64  0.000000E+00       8221.02       41.6032       10185.5 -1.000000E+10 -1.000000E+10 -1.000000E+10 -1.000000E+10 -1.000000E+10  0.000000E+00  0.000000E+00  0.000000E+00  0.000000E+00  0.000000E+00  0.000000E+00  0.000000E+00  0.000000E+00  0.000000E+00  0.000000E+00  0.000000E+00  0.000000E+00       Funtag_D
      13  5.462157E+05  5.116407E+05       34575.0       38.6826     0.0312087      0.164043      0.204746      0.226473     0.0299512    0.00952517    0.00704646      0.928654       Opttag_D
      13       56.0359       2.64225       1.00000  0.000000E+00       1.00000  0.000000E+00      0.879707       60.4510       1.00000       16.2775  0.000000E+00       2.13182  0.000000E+00       2.13182  0.000000E+00  0.000000E+00      -4.26364       56.3253       2.16394        1       Scatag_D
      13       1395.92       4701.79  0.000000E+00  0.000000E+00       2278.30  0.000000E+00       2276.42       5333.40  0.000000E+00       8343.85       41.7529       10203.6 -1.000000E+10 -1.000000E+10 -1.000000E+10 -1.000000E+10 -1.000000E+10  0.000000E+00  0.000000E+00  0.000000E+00  0.000000E+00  0.000000E+00  0.000000E+00  0.000000E+00  0.000000E+00  0.000000E+00  0.000000E+00  0.000000E+00  0.000000E+00       Funtag_D
      14  5.459002E+05  5.111083E+05       34791.9       33.2678     0.0340741      0.181331      0.204362      0.226307     0.0312935    0.00998951    0.00702088      0.934260       Opttag_D
      14       56.0070       2.61654       1.00000  0.000000E+00       1.00000  0.000000E+00      0.879450       60.5317       1.00000       16.2432  0.000000E+00       2.12879  0.000000E+00       2.12879  0.000000E+00  0.000000E+00      -4.25757       56.3253       2.16394        1       Scatag_D
      14       1385.80       4758.73  0.000000E+00  0.000000E+00       2278.01  0.000000E+00       2308.22       5352.63  0.000000E+00       8449.32       41.6957       10217.4 -1.000000E+10 -1.000000E+10 -1.000000E+10 -1.000000E+10 -1.000000E+10  0.000000E+00  0.000000E+00  0.000000E+00  0.000000E+00  0.000000E+00  0.000000E+00  0.000000E+00  0.000000E+00  0.000000E+00  0.000000E+00  0.000000E+00  0.000000E+00       Funtag_D
      15  5.456503E+05  5.106067E+05       35043.6       30.6777     0.0368583      0.198413      0.203999      0.226148     0.0325582     0.0104254    0.00701773      0.939380       Opttag_D
      15       55.9829       2.59271       1.00000  0.000000E+00       1.00000  0.000000E+00      0.879198       60.6090       1.00000       16.2128  0.000000E+00       2.12660  0.000000E+00       2.12660  0.000000E+00  0.000000E+00      -4.25320       56.3253       2.16394        1       Scatag_D
      15       1384.56       4811.04  0.000000E+00  0.000000E+00       2277.88  0.000000E+00       2329.67       5410.98  0.000000E+00       8553.82       42.2595       10233.4 -1.000000E+10 -1.000000E+10 -1.000000E+10 -1.000000E+10 -1.000000E+10  0.000000E+00  0.000000E+00  0.000000E+00  0.000000E+00  0.000000E+00  0.000000E+00  0.000000E+00  0.000000E+00  0.000000E+00  0.000000E+00  0.000000E+00  0.000000E+00       Funtag_D
      16  5.453684E+05  5.100162E+05       35352.2       31.5276     0.0401852      0.219105      0.203572      0.225976     0.0340472     0.0109352    0.00705075      0.946484       Opttag_D
      16       55.9596       2.56513       1.00000  0.000000E+00       1.00000  0.000000E+00      0.878899       60.6901       1.00000       16.1846  0.000000E+00       2.12375  0.000000E+00       2.12375  0.000000E+00  0.000000E+00      -4.24749       56.3253       2.16394        1       Scatag_D
      16       1397.62       4884.08  0.000000E+00  0.000000E+00       2277.86  0.000000E+00       2344.88       5471.96  0.000000E+00       8679.31       43.9309       10252.6 -1.000000E+10 -1.000000E+10 -1.000000E+10 -1.000000E+10 -1.000000E+10  0.000000E+00  0.000000E+00  0.000000E+00  0.000000E+00  0.000000E+00  0.000000E+00  0.000000E+00  0.000000E+00  0.000000E+00  0.000000E+00  0.000000E+00  0.000000E+00       Funtag_D
      17  5.450904E+05  5.093091E+05       35781.2       36.5311     0.0443502      0.244918      0.203076      0.225785     0.0358300     0.0114997    0.00713753      0.956788       Opttag_D
      17       55.9379       2.53257       1.00000  0.000000E+00       1.00000  0.000000E+00      0.878542       60.7748       1.00000       16.1654  0.000000E+00       2.12052  0.000000E+00       2.12052  0.000000E+00  0.000000E+00      -4.24105       56.3253       2.16394        1       Scatag_D
      17       1432.24       4991.00  0.000000E+00  0.000000E+00       2277.91  0.000000E+00       2362.91       5554.98  0.000000E+00       8836.64       47.1141       10278.4 -1.000000E+10 -1.000000E+10 -1.000000E+10 -1.000000E+10 -1.000000E+10  0.000000E+00  0.000000E+00  0.000000E+00  0.000000E+00  0.000000E+00  0.000000E+00  0.000000E+00  0.000000E+00  0.000000E+00  0.000000E+00  0.000000E+00  0.000000E+00       Funtag_D
      18  5.448530E+05  5.089559E+05       35897.1       25.8610     0.0465884      0.259090      0.202828      0.225684     0.0367207     0.0118213    0.00716580      0.957508       Opttag_D
      18       55.9314       2.51652       1.00000  0.000000E+00       1.00000  0.000000E+00      0.878353       60.8132       1.00000       16.1622  0.000000E+00       2.11972  0.000000E+00       2.11972  0.000000E+00  0.000000E+00      -4.23944       56.3253       2.16394        1       Scatag_D
      18       1443.61       4998.51  0.000000E+00  0.000000E+00       2278.33  0.000000E+00       2367.72       5557.25  0.000000E+00       8918.45       48.6259       10284.6 -1.000000E+10 -1.000000E+10 -1.000000E+10 -1.000000E+10 -1.000000E+10  0.000000E+00  0.000000E+00  0.000000E+00  0.000000E+00  0.000000E+00  0.000000E+00  0.000000E+00  0.000000E+00  0.000000E+00  0.000000E+00  0.000000E+00  0.000000E+00       Funtag_D
      19  5.446539E+05  5.086436E+05       36010.3       26.2470     0.0488816      0.273663      0.202608      0.225588     0.0375080     0.0121272    0.00717621      0.960683       Opttag_D
      19       55.9272       2.50239       1.00000  0.000000E+00       1.00000  0.000000E+00      0.878162       60.8596       1.00000       16.1588  0.000000E+00       2.11993  0.000000E+00       2.11993  0.000000E+00  0.000000E+00      -4.23986       56.3253       2.16394        1       Scatag_D
      19       1447.80       5031.72  0.000000E+00  0.000000E+00       2278.81  0.000000E+00       2373.84       5542.37  0.000000E+00       8999.52       49.2684       10287.0 -1.000000E+10 -1.000000E+10 -1.000000E+10 -1.000000E+10 -1.000000E+10  0.000000E+00  0.000000E+00  0.000000E+00  0.000000E+00  0.000000E+00  0.000000E+00  0.000000E+00  0.000000E+00  0.000000E+00  0.000000E+00  0.000000E+00  0.000000E+00       Funtag_D
      20  5.444588E+05  5.082406E+05       36218.2       31.3340     0.0523662      0.295683      0.202311      0.225451     0.0385240     0.0125493    0.00717707      0.965876       Opttag_D
      20       55.9233       2.48426       1.00000  0.000000E+00       1.00000  0.000000E+00      0.877874       60.9467       1.00000       16.1507  0.000000E+00       2.12125  0.000000E+00       2.12125  0.000000E+00  0.000000E+00      -4.24249       56.3253       2.16394        1       Scatag_D
      20       1448.15       5086.26  0.000000E+00  0.000000E+00       2279.94  0.000000E+00       2386.92       5549.19  0.000000E+00       9123.52       49.4636       10294.8 -1.000000E+10 -1.000000E+10 -1.000000E+10 -1.000000E+10 -1.000000E+10  0.000000E+00  0.000000E+00  0.000000E+00  0.000000E+00  0.000000E+00  0.000000E+00  0.000000E+00  0.000000E+00  0.000000E+00  0.000000E+00  0.000000E+00  0.000000E+00       Funtag_D
      21  5.442526E+05  5.079308E+05       36321.9       25.9556     0.0555113      0.315448      0.202073      0.225334     0.0393054     0.0128904    0.00712903      0.968885       Opttag_D
      21       55.9210       2.47044       1.00000  0.000000E+00       1.00000  0.000000E+00      0.877615       61.0411       1.00000       16.1382  0.000000E+00       2.12293  0.000000E+00       2.12293  0.000000E+00  0.000000E+00      -4.24587       56.3253       2.16394        1       Scatag_D
      21       1428.83       5118.00  0.000000E+00  0.000000E+00       2281.28  0.000000E+00       2402.04       5499.67  0.000000E+00       9236.29       49.4119       10306.4 -1.000000E+10 -1.000000E+10 -1.000000E+10 -1.000000E+10 -1.000000E+10  0.000000E+00  0.000000E+00  0.000000E+00  0.000000E+00  0.000000E+00  0.000000E+00  0.000000E+00  0.000000E+00  0.000000E+00  0.000000E+00  0.000000E+00  0.000000E+00       Funtag_D
      22  5.440550E+05  5.076384E+05       36416.6       23.4757     0.0586451      0.335196      0.201844      0.225213     0.0400425     0.0132047    0.00708186      0.973120       Opttag_D
      22       55.9208       2.45750       1.00000  0.000000E+00       1.00000  0.000000E+00      0.877356       61.1353       1.00000       16.1279  0.000000E+00       2.12406  0.000000E+00       2.12406  0.000000E+00  0.000000E+00      -4.24812       56.3253       2.16394        1       Scatag_D
      22       1409.98       5162.84  0.000000E+00  0.000000E+00       2282.66  0.000000E+00       2418.50       5427.60  0.000000E+00       9346.78       49.6325       10318.6 -1.000000E+10 -1.000000E+10 -1.000000E+10 -1.000000E+10 -1.000000E+10  0.000000E+00  0.000000E+00  0.000000E+00  0.000000E+00  0.000000E+00  0.000000E+00  0.000000E+00  0.000000E+00  0.000000E+00  0.000000E+00  0.000000E+00  0.000000E+00       Funtag_D
      23  5.438867E+05  5.072325E+05       36654.2       30.6430     0.0627046      0.360782      0.201542      0.225057     0.0410662     0.0135847    0.00708074      0.980338       Opttag_D
      23       55.9242       2.43986       1.00000  0.000000E+00       1.00000  0.000000E+00      0.877027       61.2376       1.00000       16.1248  0.000000E+00       2.12372  0.000000E+00       2.12372  0.000000E+00  0.000000E+00      -4.24745       56.3253       2.16394        1       Scatag_D
      23       1409.54       5239.72  0.000000E+00  0.000000E+00       2284.65  0.000000E+00       2441.86       5400.52  0.000000E+00       9489.43       50.9619       10337.5 -1.000000E+10 -1.000000E+10 -1.000000E+10 -1.000000E+10 -1.000000E+10  0.000000E+00  0.000000E+00  0.000000E+00  0.000000E+00  0.000000E+00  0.000000E+00  0.000000E+00  0.000000E+00  0.000000E+00  0.000000E+00  0.000000E+00  0.000000E+00       Funtag_D
      24  5.437365E+05  5.069607E+05       36775.8       22.8011     0.0652473      0.376969      0.201344      0.224960     0.0417514     0.0138045    0.00709134      0.981448       Opttag_D
      24       55.9293       2.42836       1.00000  0.000000E+00       1.00000  0.000000E+00      0.876827       61.2880       1.00000       16.1291  0.000000E+00       2.12213  0.000000E+00       2.12213  0.000000E+00  0.000000E+00      -4.24425       56.3253       2.16394        1       Scatag_D
      24       1413.76       5251.59  0.000000E+00  0.000000E+00       2286.05  0.000000E+00       2456.93       5392.92  0.000000E+00       9574.60       52.1220       10347.8 -1.000000E+10 -1.000000E+10 -1.000000E+10 -1.000000E+10 -1.000000E+10  0.000000E+00  0.000000E+00  0.000000E+00  0.000000E+00  0.000000E+00  0.000000E+00  0.000000E+00  0.000000E+00  0.000000E+00  0.000000E+00  0.000000E+00  0.000000E+00       Funtag_D
      25  5.435927E+05  5.067291E+05       36863.6       19.9889     0.0670280      0.388661      0.201182      0.224879     0.0423353     0.0139849    0.00712784      0.982962       Opttag_D
      25       55.9365       2.41885       1.00000  0.000000E+00       1.00000  0.000000E+00      0.876693       61.3018       1.00000       16.1403  0.000000E+00       2.11906  0.000000E+00       2.11906  0.000000E+00  0.000000E+00      -4.23812       56.3253       2.16394        1       Scatag_D
      25       1428.35       5267.80  0.000000E+00  0.000000E+00       2286.76  0.000000E+00       2467.09       5382.42  0.000000E+00       9626.56       53.1777       10351.5 -1.000000E+10 -1.000000E+10 -1.000000E+10 -1.000000E+10 -1.000000E+10  0.000000E+00  0.000000E+00  0.000000E+00  0.000000E+00  0.000000E+00  0.000000E+00  0.000000E+00  0.000000E+00  0.000000E+00  0.000000E+00  0.000000E+00  0.000000E+00       Funtag_D
      26  5.434083E+05  5.063432E+05       37065.1       26.9851     0.0703616      0.411483      0.200910      0.224747     0.0433084     0.0143134    0.00720956      0.987510       Opttag_D
      26       55.9535       2.40358       1.00000  0.000000E+00       1.00000  0.000000E+00      0.876451       61.3358       1.00000       16.1608  0.000000E+00       2.11306  0.000000E+00       2.11306  0.000000E+00  0.000000E+00      -4.22612       56.3253       2.16394        1       Scatag_D
      26       1461.29       5316.66  0.000000E+00  0.000000E+00       2288.45  0.000000E+00       2488.16       5378.69  0.000000E+00       9721.34       54.0325       10356.5 -1.000000E+10 -1.000000E+10 -1.000000E+10 -1.000000E+10 -1.000000E+10  0.000000E+00  0.000000E+00  0.000000E+00  0.000000E+00  0.000000E+00  0.000000E+00  0.000000E+00  0.000000E+00  0.000000E+00  0.000000E+00  0.000000E+00  0.000000E+00       Funtag_D
      27  5.432862E+05  5.058988E+05       37387.4       38.4718     0.0751102      0.446419      0.200584      0.224571     0.0444288     0.0147052    0.00730100      0.995202       Opttag_D
      27       55.9816       2.38677       1.00000  0.000000E+00       1.00000  0.000000E+00      0.876123       61.4094       1.00000       16.1866  0.000000E+00       2.10498  0.000000E+00       2.10498  0.000000E+00  0.000000E+00      -4.20996       56.3253       2.16394        1       Scatag_D
      27       1498.59       5399.80  0.000000E+00  0.000000E+00       2291.53  0.000000E+00       2521.72       5398.33  0.000000E+00       9856.83       54.9204       10365.7 -1.000000E+10 -1.000000E+10 -1.000000E+10 -1.000000E+10 -1.000000E+10  0.000000E+00  0.000000E+00  0.000000E+00  0.000000E+00  0.000000E+00  0.000000E+00  0.000000E+00  0.000000E+00  0.000000E+00  0.000000E+00  0.000000E+00  0.000000E+00       Funtag_D
      28  5.431308E+05  5.058952E+05       37235.5       19.8344     0.0753015      0.448868      0.200578      0.224554     0.0444378     0.0147767    0.00725132      0.990545       Opttag_D
      28       55.9870       2.38717       1.00000  0.000000E+00       1.00000  0.000000E+00      0.876114       61.4160       1.00000       16.1881  0.000000E+00       2.10310  0.000000E+00       2.10310  0.000000E+00  0.000000E+00      -4.20620       56.3253       2.16394        1       Scatag_D
      28       1478.27       5349.40  0.000000E+00  0.000000E+00       2291.44  0.000000E+00       2521.76       5332.30  0.000000E+00       9853.11       54.3241       10354.9 -1.000000E+10 -1.000000E+10 -1.000000E+10 -1.000000E+10 -1.000000E+10  0.000000E+00  0.000000E+00  0.000000E+00  0.000000E+00  0.000000E+00  0.000000E+00  0.000000E+00  0.000000E+00  0.000000E+00  0.000000E+00  0.000000E+00  0.000000E+00       Funtag_D
      29  5.430008E+05  5.058411E+05       37159.7       18.1962     0.0764104      0.458477      0.200524      0.224504     0.0445742     0.0149422    0.00720812      0.990785       Opttag_D
      29       56.0003       2.38613       1.00000  0.000000E+00       1.00000  0.000000E+00      0.876047       61.4514       1.00000       16.1917  0.000000E+00       2.09893  0.000000E+00       2.09893  0.000000E+00  0.000000E+00      -4.19786       56.3253       2.16394        1       Scatag_D
      29       1460.70       5351.99  0.000000E+00  0.000000E+00       2292.12  0.000000E+00       2527.51       5257.58  0.000000E+00       9872.86       53.8742       10343.1 -1.000000E+10 -1.000000E+10 -1.000000E+10 -1.000000E+10 -1.000000E+10  0.000000E+00  0.000000E+00  0.000000E+00  0.000000E+00  0.000000E+00  0.000000E+00  0.000000E+00  0.000000E+00  0.000000E+00  0.000000E+00  0.000000E+00  0.000000E+00       Funtag_D
      30  5.428827E+05  5.056942E+05       37188.5       21.9214     0.0787565      0.477268      0.200399      0.224405     0.0449447     0.0151780    0.00719653      0.992721       Opttag_D
      30       56.0245       2.38208       1.00000  0.000000E+00       1.00000  0.000000E+00      0.875906       61.5134       1.00000       16.2029  0.000000E+00       2.09087  0.000000E+00       2.09087  0.000000E+00  0.000000E+00      -4.18174       56.3253       2.16394        1       Scatag_D
      30       1456.01       5372.92  0.000000E+00  0.000000E+00       2293.86  0.000000E+00       2541.71       5209.12  0.000000E+00       9924.63       54.3342       10335.9 -1.000000E+10 -1.000000E+10 -1.000000E+10 -1.000000E+10 -1.000000E+10  0.000000E+00  0.000000E+00  0.000000E+00  0.000000E+00  0.000000E+00  0.000000E+00  0.000000E+00  0.000000E+00  0.000000E+00  0.000000E+00  0.000000E+00  0.000000E+00       Funtag_D
      31  5.427611E+05  5.054630E+05       37298.1       30.1863     0.0828791      0.510057      0.200197      0.224237     0.0455276     0.0155621    0.00718717      0.997201       Opttag_D
      31       56.0719       2.37662       1.00000  0.000000E+00       1.00000  0.000000E+00      0.875684       61.6257       1.00000       16.2246  0.000000E+00       2.07438  0.000000E+00       2.07438  0.000000E+00  0.000000E+00      -4.14876       56.3253       2.16394        1       Scatag_D
      31       1452.23       5421.52  0.000000E+00  0.000000E+00       2297.13  0.000000E+00       2566.96       5159.10  0.000000E+00       10012.0       55.6293       10333.6 -1.000000E+10 -1.000000E+10 -1.000000E+10 -1.000000E+10 -1.000000E+10  0.000000E+00  0.000000E+00  0.000000E+00  0.000000E+00  0.000000E+00  0.000000E+00  0.000000E+00  0.000000E+00  0.000000E+00  0.000000E+00  0.000000E+00  0.000000E+00       Funtag_D
      32  5.426400E+05  5.053965E+05       37243.5       16.8917     0.0837864      0.517823      0.200142      0.224203     0.0456954     0.0156232    0.00718415      0.994936       Opttag_D
      32       56.0866       2.37531       1.00000  0.000000E+00       1.00000  0.000000E+00      0.875649       61.6484       1.00000       16.2306  0.000000E+00       2.06819  0.000000E+00       2.06819  0.000000E+00  0.000000E+00      -4.13638       56.3253       2.16394        1       Scatag_D
      32       1451.01       5396.92  0.000000E+00  0.000000E+00       2298.05  0.000000E+00       2569.89       5117.47  0.000000E+00       10023.3       55.9659       10330.9 -1.000000E+10 -1.000000E+10 -1.000000E+10 -1.000000E+10 -1.000000E+10  0.000000E+00  0.000000E+00  0.000000E+00  0.000000E+00  0.000000E+00  0.000000E+00  0.000000E+00  0.000000E+00  0.000000E+00  0.000000E+00  0.000000E+00  0.000000E+00       Funtag_D
      33  5.425231E+05  5.053130E+05       37210.1       16.6309     0.0846853      0.526380      0.200068      0.224171     0.0459057     0.0156900    0.00720030      0.994702       Opttag_D
      33       56.1072       2.37405       1.00000  0.000000E+00       1.00000  0.000000E+00      0.875632       61.6686       1.00000       16.2371  0.000000E+00       2.05871  0.000000E+00       2.05871  0.000000E+00  0.000000E+00      -4.11741       56.3253       2.16394        1       Scatag_D
      33       1457.54       5394.39  0.000000E+00  0.000000E+00       2298.95  0.000000E+00       2569.47       5085.18  0.000000E+00       10023.2       56.0817       10325.3 -1.000000E+10 -1.000000E+10 -1.000000E+10 -1.000000E+10 -1.000000E+10  0.000000E+00  0.000000E+00  0.000000E+00  0.000000E+00  0.000000E+00  0.000000E+00  0.000000E+00  0.000000E+00  0.000000E+00  0.000000E+00  0.000000E+00  0.000000E+00       Funtag_D
      34  5.424186E+05  5.052033E+05       37215.3       19.5990     0.0860916      0.539222      0.199973      0.224136     0.0461824     0.0157631    0.00722517      0.995075       Opttag_D
      34       56.1373       2.37267       1.00000  0.000000E+00       1.00000  0.000000E+00      0.875602       61.7012       1.00000       16.2466  0.000000E+00       2.04582  0.000000E+00       2.04582  0.000000E+00  0.000000E+00      -4.09164       56.3253       2.16394        1       Scatag_D
      34       1467.62       5398.44  0.000000E+00  0.000000E+00       2300.45  0.000000E+00       2570.12       5073.85  0.000000E+00       10027.8       55.6912       10321.3 -1.000000E+10 -1.000000E+10 -1.000000E+10 -1.000000E+10 -1.000000E+10  0.000000E+00  0.000000E+00  0.000000E+00  0.000000E+00  0.000000E+00  0.000000E+00  0.000000E+00  0.000000E+00  0.000000E+00  0.000000E+00  0.000000E+00  0.000000E+00       Funtag_D
      35  5.423393E+05  5.050070E+05       37332.3       35.4264     0.0891973      0.566408      0.199794      0.224082     0.0466773     0.0158203    0.00730012      0.998930       Opttag_D
      35       56.2024       2.37131       1.00000  0.000000E+00       1.00000  0.000000E+00      0.875534       61.7789       1.00000       16.2661  0.000000E+00       2.01954  0.000000E+00       2.01954  0.000000E+00  0.000000E+00      -4.03907       56.3253       2.16394        1       Scatag_D
      35       1498.23       5440.34  0.000000E+00  0.000000E+00       2303.81  0.000000E+00       2572.20       5095.66  0.000000E+00       10043.3       54.7931       10323.9 -1.000000E+10 -1.000000E+10 -1.000000E+10 -1.000000E+10 -1.000000E+10  0.000000E+00  0.000000E+00  0.000000E+00  0.000000E+00  0.000000E+00  0.000000E+00  0.000000E+00  0.000000E+00  0.000000E+00  0.000000E+00  0.000000E+00  0.000000E+00       Funtag_D
      36  5.422230E+05  5.049882E+05       37234.8       15.4231     0.0894981      0.569314      0.199774      0.224077     0.0467247     0.0158338    0.00725802      0.995361       Opttag_D
      36       56.2107       2.37142       1.00000  0.000000E+00       1.00000  0.000000E+00      0.875531       61.7878       1.00000       16.2671  0.000000E+00       2.01660  0.000000E+00       2.01660  0.000000E+00  0.000000E+00      -4.03319       56.3253       2.16394        1       Scatag_D
      36       1481.00       5401.53  0.000000E+00  0.000000E+00       2304.26  0.000000E+00       2568.95       5064.18  0.000000E+00       10040.7       54.4624       10319.7 -1.000000E+10 -1.000000E+10 -1.000000E+10 -1.000000E+10 -1.000000E+10  0.000000E+00  0.000000E+00  0.000000E+00  0.000000E+00  0.000000E+00  0.000000E+00  0.000000E+00  0.000000E+00  0.000000E+00  0.000000E+00  0.000000E+00  0.000000E+00       Funtag_D
      37  5.421607E+05  5.049678E+05       37192.9       12.8869     0.0898690      0.572779      0.199754      0.224070     0.0467761     0.0158356    0.00723638      0.995189       Opttag_D
      37       56.2204       2.37158       1.00000  0.000000E+00       1.00000  0.000000E+00      0.875524       61.7998       1.00000       16.2677  0.000000E+00       2.01344  0.000000E+00       2.01344  0.000000E+00  0.000000E+00      -4.02688       56.3253       2.16394        1       Scatag_D
      37       1472.18       5399.67  0.000000E+00  0.000000E+00       2304.84  0.000000E+00       2564.47       5043.78  0.000000E+00       10038.5       54.2056       10315.3 -1.000000E+10 -1.000000E+10 -1.000000E+10 -1.000000E+10 -1.000000E+10  0.000000E+00  0.000000E+00  0.000000E+00  0.000000E+00  0.000000E+00  0.000000E+00  0.000000E+00  0.000000E+00  0.000000E+00  0.000000E+00  0.000000E+00  0.000000E+00       Funtag_D
      38  5.420877E+05  5.049166E+05       37171.1       14.6415     0.0910021      0.582623      0.199696      0.224041     0.0469054     0.0158454    0.00721953      0.995330       Opttag_D
      38       56.2478       2.37230       1.00000  0.000000E+00       1.00000  0.000000E+00      0.875506       61.8353       1.00000       16.2721  0.000000E+00       2.00399  0.000000E+00       2.00399  0.000000E+00  0.000000E+00      -4.00798       56.3253       2.16394        1       Scatag_D
      38       1465.33       5401.20  0.000000E+00  0.000000E+00       2306.37  0.000000E+00       2558.17       5037.46  0.000000E+00       10038.6       54.1037       10309.9 -1.000000E+10 -1.000000E+10 -1.000000E+10 -1.000000E+10 -1.000000E+10  0.000000E+00  0.000000E+00  0.000000E+00  0.000000E+00  0.000000E+00  0.000000E+00  0.000000E+00  0.000000E+00  0.000000E+00  0.000000E+00  0.000000E+00  0.000000E+00       Funtag_D
      39  5.420286E+05  5.048357E+05       37193.0       33.6072     0.0931109      0.600104      0.199598      0.223963     0.0471094     0.0158790    0.00721642      0.998101       Opttag_D
      39       56.3015       2.37439       1.00000  0.000000E+00       1.00000  0.000000E+00      0.875493       61.8984       1.00000       16.2807  0.000000E+00       1.98635  0.000000E+00       1.98635  0.000000E+00  0.000000E+00      -3.97270       56.3253       2.16394        1       Scatag_D
      39       1464.07       5431.31  0.000000E+00  0.000000E+00       2309.36  0.000000E+00       2546.53       5050.55  0.000000E+00       10038.7       54.4069       10298.1 -1.000000E+10 -1.000000E+10 -1.000000E+10 -1.000000E+10 -1.000000E+10  0.000000E+00  0.000000E+00  0.000000E+00  0.000000E+00  0.000000E+00  0.000000E+00  0.000000E+00  0.000000E+00  0.000000E+00  0.000000E+00  0.000000E+00  0.000000E+00       Funtag_D
      40  5.419304E+05  5.048050E+05       37125.4       15.6014     0.0940713      0.607695      0.199560      0.223929     0.0471868     0.0158749    0.00719467      0.994952       Opttag_D
      40       56.3252       2.37551       1.00000  0.000000E+00       1.00000  0.000000E+00      0.875487       61.9270       1.00000       16.2858  0.000000E+00       1.97870  0.000000E+00       1.97870  0.000000E+00  0.000000E+00      -3.95741       56.3253       2.16394        1       Scatag_D
      40       1455.26       5397.10  0.000000E+00  0.000000E+00       2310.70  0.000000E+00       2543.31       5030.63  0.000000E+00       10040.2       54.4730       10293.7 -1.000000E+10 -1.000000E+10 -1.000000E+10 -1.000000E+10 -1.000000E+10  0.000000E+00  0.000000E+00  0.000000E+00  0.000000E+00  0.000000E+00  0.000000E+00  0.000000E+00  0.000000E+00  0.000000E+00  0.000000E+00  0.000000E+00  0.000000E+00       Funtag_D
      41  5.418677E+05  5.047754E+05       37092.4       11.5159     0.0948862      0.618960      0.199521      0.223890     0.0472615     0.0158966    0.00719439      0.994164       Opttag_D
      41       56.3469       2.37645       1.00000  0.000000E+00       1.00000  0.000000E+00      0.875490       61.9484       1.00000       16.2903  0.000000E+00       1.97206  0.000000E+00       1.97206  0.000000E+00  0.000000E+00      -3.94413       56.3253       2.16394        1       Scatag_D
      41       1455.15       5388.55  0.000000E+00  0.000000E+00       2311.86  0.000000E+00       2540.11       5014.69  0.000000E+00       10039.8       54.4978       10287.7 -1.000000E+10 -1.000000E+10 -1.000000E+10 -1.000000E+10 -1.000000E+10  0.000000E+00  0.000000E+00  0.000000E+00  0.000000E+00  0.000000E+00  0.000000E+00  0.000000E+00  0.000000E+00  0.000000E+00  0.000000E+00  0.000000E+00  0.000000E+00       Funtag_D
      42  5.418016E+05  5.047316E+05       37070.0       13.7197     0.0961099      0.644361      0.199467      0.223839     0.0473718     0.0159230    0.00719889      0.993658       Opttag_D
      42       56.3799       2.37788       1.00000  0.000000E+00       1.00000  0.000000E+00      0.875500       61.9775       1.00000       16.2970  0.000000E+00       1.96244  0.000000E+00       1.96244  0.000000E+00  0.000000E+00      -3.92488       56.3253       2.16394        1       Scatag_D
      42       1456.97       5383.06  0.000000E+00  0.000000E+00       2313.68  0.000000E+00       2535.39       5008.94  0.000000E+00       10038.6       54.2333       10279.1 -1.000000E+10 -1.000000E+10 -1.000000E+10 -1.000000E+10 -1.000000E+10  0.000000E+00  0.000000E+00  0.000000E+00  0.000000E+00  0.000000E+00  0.000000E+00  0.000000E+00  0.000000E+00  0.000000E+00  0.000000E+00  0.000000E+00  0.000000E+00       Funtag_D
      43  5.417223E+05  5.046588E+05       37063.5       22.9129     0.0981900      0.688691      0.199386      0.223767     0.0475555     0.0159925    0.00720936      0.993257       Opttag_D
      43       56.4345       2.38010       1.00000  0.000000E+00       1.00000  0.000000E+00      0.875514       62.0203       1.00000       16.3090  0.000000E+00       1.94775  0.000000E+00       1.94775  0.000000E+00  0.000000E+00      -3.89550       56.3253       2.16394        1       Scatag_D
      43       1461.21       5378.72  0.000000E+00  0.000000E+00       2316.79  0.000000E+00       2528.90       5020.21  0.000000E+00       10036.6       53.3769       10267.7 -1.000000E+10 -1.000000E+10 -1.000000E+10 -1.000000E+10 -1.000000E+10  0.000000E+00  0.000000E+00  0.000000E+00  0.000000E+00  0.000000E+00  0.000000E+00  0.000000E+00  0.000000E+00  0.000000E+00  0.000000E+00  0.000000E+00  0.000000E+00       Funtag_D
      44  5.416454E+05  5.046014E+05       37044.0       14.4007      0.100151      0.730021      0.199312      0.223718     0.0477000     0.0160065    0.00720905      0.991533       Opttag_D
      44       56.4844       2.38246       1.00000  0.000000E+00       1.00000  0.000000E+00      0.875527       62.0583       1.00000       16.3217  0.000000E+00       1.93489  0.000000E+00       1.93489  0.000000E+00  0.000000E+00      -3.86978       56.3253       2.16394        1       Scatag_D
      44       1461.08       5360.06  0.000000E+00  0.000000E+00       2319.80  0.000000E+00       2524.36       5029.29  0.000000E+00       10036.7       52.7012       10259.9 -1.000000E+10 -1.000000E+10 -1.000000E+10 -1.000000E+10 -1.000000E+10  0.000000E+00  0.000000E+00  0.000000E+00  0.000000E+00  0.000000E+00  0.000000E+00  0.000000E+00  0.000000E+00  0.000000E+00  0.000000E+00  0.000000E+00  0.000000E+00       Funtag_D
      45  5.415949E+05  5.045750E+05       37019.9       10.4548      0.100464      0.739626      0.199292      0.223693     0.0477668     0.0160672    0.00719993      0.990903       Opttag_D
      45       56.4924       2.38205       1.00000  0.000000E+00       1.00000  0.000000E+00      0.875526       62.0572       1.00000       16.3247  0.000000E+00       1.93345  0.000000E+00       1.93345  0.000000E+00  0.000000E+00      -3.86689       56.3253       2.16394        1       Scatag_D
      45       1457.39       5353.26  0.000000E+00  0.000000E+00       2320.26  0.000000E+00       2522.89       5022.60  0.000000E+00       10033.8       52.5342       10257.2 -1.000000E+10 -1.000000E+10 -1.000000E+10 -1.000000E+10 -1.000000E+10  0.000000E+00  0.000000E+00  0.000000E+00  0.000000E+00  0.000000E+00  0.000000E+00  0.000000E+00  0.000000E+00  0.000000E+00  0.000000E+00  0.000000E+00  0.000000E+00       Funtag_D
      46  5.415167E+05  5.045103E+05       37006.4       14.2149      0.101279      0.764735      0.199244      0.223633     0.0479299     0.0162244    0.00718946      0.990202       Opttag_D
      46       56.5127       2.38109       1.00000  0.000000E+00       1.00000  0.000000E+00      0.875520       62.0552       1.00000       16.3339  0.000000E+00       1.93024  0.000000E+00       1.93024  0.000000E+00  0.000000E+00      -3.86049       56.3253       2.16394        1       Scatag_D
      46       1453.15       5345.69  0.000000E+00  0.000000E+00       2321.48  0.000000E+00       2519.65       5035.29  0.000000E+00       10027.3       52.3715       10251.5 -1.000000E+10 -1.000000E+10 -1.000000E+10 -1.000000E+10 -1.000000E+10  0.000000E+00  0.000000E+00  0.000000E+00  0.000000E+00  0.000000E+00  0.000000E+00  0.000000E+00  0.000000E+00  0.000000E+00  0.000000E+00  0.000000E+00  0.000000E+00       Funtag_D
      47  5.414696E+05  5.044397E+05       37030.0       22.9064      0.102813      0.804574      0.199186      0.223558     0.0481078     0.0163522    0.00719000      0.989754       Opttag_D
      47       56.5498       2.38146       1.00000  0.000000E+00       1.00000  0.000000E+00      0.875514       62.0665       1.00000       16.3519  0.000000E+00       1.92277  0.000000E+00       1.92277  0.000000E+00  0.000000E+00      -3.84554       56.3253       2.16394        1       Scatag_D
      47       1453.37       5340.85  0.000000E+00  0.000000E+00       2323.80  0.000000E+00       2516.46       5076.13  0.000000E+00       10023.3       52.3486       10243.7 -1.000000E+10 -1.000000E+10 -1.000000E+10 -1.000000E+10 -1.000000E+10  0.000000E+00  0.000000E+00  0.000000E+00  0.000000E+00  0.000000E+00  0.000000E+00  0.000000E+00  0.000000E+00  0.000000E+00  0.000000E+00  0.000000E+00  0.000000E+00       Funtag_D
      48  5.414050E+05  5.044156E+05       36989.4       13.5937      0.103317      0.818737      0.199169      0.223525     0.0481686     0.0164246    0.00718505      0.989008       Opttag_D
      48       56.5617       2.38144       1.00000  0.000000E+00       1.00000  0.000000E+00      0.875511       62.0669       1.00000       16.3600  0.000000E+00       1.92083  0.000000E+00       1.92083  0.000000E+00  0.000000E+00      -3.84166       56.3253       2.16394        1       Scatag_D
      48       1451.37       5332.80  0.000000E+00  0.000000E+00       2324.67  0.000000E+00       2515.46       5052.33  0.000000E+00       10022.1       52.4413       10238.2 -1.000000E+10 -1.000000E+10 -1.000000E+10 -1.000000E+10 -1.000000E+10  0.000000E+00  0.000000E+00  0.000000E+00  0.000000E+00  0.000000E+00  0.000000E+00  0.000000E+00  0.000000E+00  0.000000E+00  0.000000E+00  0.000000E+00  0.000000E+00       Funtag_D
      49  5.413302E+05  5.043755E+05       36954.7       10.9281      0.104558      0.851053      0.199139      0.223468     0.0482697     0.0165242    0.00718458      0.988538       Opttag_D
      49       56.5905       2.38222       1.00000  0.000000E+00       1.00000  0.000000E+00      0.875504       62.0731       1.00000       16.3796  0.000000E+00       1.91551  0.000000E+00       1.91551  0.000000E+00  0.000000E+00      -3.83102       56.3253       2.16394        1       Scatag_D
      49       1451.18       5327.73  0.000000E+00  0.000000E+00       2326.83  0.000000E+00       2513.70       5035.95  0.000000E+00       10021.9       52.5842       10224.8 -1.000000E+10 -1.000000E+10 -1.000000E+10 -1.000000E+10 -1.000000E+10  0.000000E+00  0.000000E+00  0.000000E+00  0.000000E+00  0.000000E+00  0.000000E+00  0.000000E+00  0.000000E+00  0.000000E+00  0.000000E+00  0.000000E+00  0.000000E+00       Funtag_D
      50  5.412788E+05  5.043496E+05       36929.2       12.2320      0.105757      0.881745      0.199118      0.223427     0.0483350     0.0165912    0.00718056      0.988345       Opttag_D
      50       56.6178       2.38347       1.00000  0.000000E+00       1.00000  0.000000E+00      0.875498       62.0797       1.00000       16.3979  0.000000E+00       1.91065  0.000000E+00       1.91065  0.000000E+00  0.000000E+00      -3.82130       56.3253       2.16394        1       Scatag_D
      50       1449.56       5325.65  0.000000E+00  0.000000E+00       2328.98  0.000000E+00       2512.95       5024.06  0.000000E+00       10023.5       52.4340       10212.1 -1.000000E+10 -1.000000E+10 -1.000000E+10 -1.000000E+10 -1.000000E+10  0.000000E+00  0.000000E+00  0.000000E+00  0.000000E+00  0.000000E+00  0.000000E+00  0.000000E+00  0.000000E+00  0.000000E+00  0.000000E+00  0.000000E+00  0.000000E+00       Funtag_D
      51  5.412390E+05  5.043123E+05       36926.7       25.4436      0.108737      0.956181      0.199099      0.223343     0.0484291     0.0167095    0.00718305      0.988418       Opttag_D
      51       56.6826       2.38742       1.00000  0.000000E+00       1.00000  0.000000E+00      0.875477       62.0987       1.00000       16.4451  0.000000E+00       1.89955  0.000000E+00       1.89955  0.000000E+00  0.000000E+00      -3.79911       56.3253       2.16394        1       Scatag_D
      51       1450.56       5326.44  0.000000E+00  0.000000E+00       2334.38  0.000000E+00       2513.52       5031.88  0.000000E+00       10033.1       52.0691       10184.7 -1.000000E+10 -1.000000E+10 -1.000000E+10 -1.000000E+10 -1.000000E+10  0.000000E+00  0.000000E+00  0.000000E+00  0.000000E+00  0.000000E+00  0.000000E+00  0.000000E+00  0.000000E+00  0.000000E+00  0.000000E+00  0.000000E+00  0.000000E+00       Funtag_D
      52  5.411678E+05  5.043077E+05       36860.1       10.4091      0.108821      0.959337      0.199097      0.223341     0.0484406     0.0167176    0.00716839      0.987871       Opttag_D
      52       56.6837       2.38729       1.00000  0.000000E+00       1.00000  0.000000E+00      0.875472       62.0959       1.00000       16.4465  0.000000E+00       1.89943  0.000000E+00       1.89943  0.000000E+00  0.000000E+00      -3.79886       56.3253       2.16394        1       Scatag_D
      52       1444.65       5320.55  0.000000E+00  0.000000E+00       2334.68  0.000000E+00       2513.43       4980.16  0.000000E+00       10034.1       51.9665       10180.6 -1.000000E+10 -1.000000E+10 -1.000000E+10 -1.000000E+10 -1.000000E+10  0.000000E+00  0.000000E+00  0.000000E+00  0.000000E+00  0.000000E+00  0.000000E+00  0.000000E+00  0.000000E+00  0.000000E+00  0.000000E+00  0.000000E+00  0.000000E+00       Funtag_D
      53  5.411376E+05  5.042991E+05       36838.5       9.00819      0.108776      0.959782      0.199094      0.223343     0.0484623     0.0167342    0.00716061      0.987760       Opttag_D
      53       56.6816       2.38676       1.00000  0.000000E+00       1.00000  0.000000E+00      0.875464       62.0920       1.00000       16.4464  0.000000E+00       1.89972  0.000000E+00       1.89972  0.000000E+00  0.000000E+00      -3.79944       56.3253       2.16394        1       Scatag_D
      53       1441.51       5319.35  0.000000E+00  0.000000E+00       2334.73  0.000000E+00       2513.38       4965.41  0.000000E+00       10034.5       51.9337       10177.7 -1.000000E+10 -1.000000E+10 -1.000000E+10 -1.000000E+10 -1.000000E+10  0.000000E+00  0.000000E+00  0.000000E+00  0.000000E+00  0.000000E+00  0.000000E+00  0.000000E+00  0.000000E+00  0.000000E+00  0.000000E+00  0.000000E+00  0.000000E+00       Funtag_D
      54  5.410846E+05  5.042625E+05       36822.1       11.8435      0.109554      0.981966      0.199077      0.223323     0.0485545     0.0167961    0.00715724      0.987639       Opttag_D
      54       56.6947       2.38628       1.00000  0.000000E+00       1.00000  0.000000E+00      0.875436       62.0876       1.00000       16.4604  0.000000E+00       1.89729  0.000000E+00       1.89729  0.000000E+00  0.000000E+00      -3.79458       56.3253       2.16394        1       Scatag_D
      54       1440.15       5318.05  0.000000E+00  0.000000E+00       2336.34  0.000000E+00       2514.33       4953.68  0.000000E+00       10039.7       51.9715       10167.8 -1.000000E+10 -1.000000E+10 -1.000000E+10 -1.000000E+10 -1.000000E+10  0.000000E+00  0.000000E+00  0.000000E+00  0.000000E+00  0.000000E+00  0.000000E+00  0.000000E+00  0.000000E+00  0.000000E+00  0.000000E+00  0.000000E+00  0.000000E+00       Funtag_D
      55  5.410507E+05  5.041691E+05       36881.7       27.0535      0.111519       1.03416      0.199033      0.223276     0.0487902     0.0169102    0.00717847      0.988379       Opttag_D
      55       56.7277       2.38510       1.00000  0.000000E+00       1.00000  0.000000E+00      0.875378       62.0802       1.00000       16.4964  0.000000E+00       1.89066  0.000000E+00       1.89066  0.000000E+00  0.000000E+00      -3.78132       56.3253       2.16394        1       Scatag_D
      55       1448.71       5326.02  0.000000E+00  0.000000E+00       2339.98  0.000000E+00       2518.40       4989.26  0.000000E+00       10052.8       52.1546       10154.4 -1.000000E+10 -1.000000E+10 -1.000000E+10 -1.000000E+10 -1.000000E+10  0.000000E+00  0.000000E+00  0.000000E+00  0.000000E+00  0.000000E+00  0.000000E+00  0.000000E+00  0.000000E+00  0.000000E+00  0.000000E+00  0.000000E+00  0.000000E+00       Funtag_D
      56  5.409851E+05  5.041435E+05       36841.6       12.5277      0.112232       1.05183      0.199021      0.223257     0.0488545     0.0169366    0.00718353      0.987899       Opttag_D
      56       56.7398       2.38495       1.00000  0.000000E+00       1.00000  0.000000E+00      0.875361       62.0793       1.00000       16.5091  0.000000E+00       1.88847  0.000000E+00       1.88847  0.000000E+00  0.000000E+00      -3.77694       56.3253       2.16394        1       Scatag_D
      56       1450.76       5320.85  0.000000E+00  0.000000E+00       2341.14  0.000000E+00       2519.90       4948.78  0.000000E+00       10057.2       52.2017       10150.8 -1.000000E+10 -1.000000E+10 -1.000000E+10 -1.000000E+10 -1.000000E+10  0.000000E+00  0.000000E+00  0.000000E+00  0.000000E+00  0.000000E+00  0.000000E+00  0.000000E+00  0.000000E+00  0.000000E+00  0.000000E+00  0.000000E+00  0.000000E+00       Funtag_D
      57  5.409460E+05  5.041011E+05       36844.8       8.80630      0.113231       1.07618      0.198997      0.223231     0.0489614     0.0169692    0.00719630      0.987878       Opttag_D
      57       56.7565       2.38447       1.00000  0.000000E+00       1.00000  0.000000E+00      0.875339       62.0773       1.00000       16.5274  0.000000E+00       1.88528  0.000000E+00       1.88528  0.000000E+00  0.000000E+00      -3.77057       56.3253       2.16394        1       Scatag_D
      57       1455.92       5320.62  0.000000E+00  0.000000E+00       2342.66  0.000000E+00       2521.87       4942.99  0.000000E+00       10062.7       52.2241       10145.8 -1.000000E+10 -1.000000E+10 -1.000000E+10 -1.000000E+10 -1.000000E+10  0.000000E+00  0.000000E+00  0.000000E+00  0.000000E+00  0.000000E+00  0.000000E+00  0.000000E+00  0.000000E+00  0.000000E+00  0.000000E+00  0.000000E+00  0.000000E+00       Funtag_D
      58  5.409076E+05  5.040395E+05       36868.1       9.71990      0.114596       1.10951      0.198963      0.223200     0.0491168     0.0170197    0.00721348      0.988003       Opttag_D
      58       56.7784       2.38357       1.00000  0.000000E+00       1.00000  0.000000E+00      0.875307       62.0728       1.00000       16.5528  0.000000E+00       1.88112  0.000000E+00       1.88112  0.000000E+00  0.000000E+00      -3.76223       56.3253       2.16394        1       Scatag_D
      58       1462.88       5321.97  0.000000E+00  0.000000E+00       2344.66  0.000000E+00       2525.40       4951.35  0.000000E+00       10070.7       52.1633       10139.0 -1.000000E+10 -1.000000E+10 -1.000000E+10 -1.000000E+10 -1.000000E+10  0.000000E+00  0.000000E+00  0.000000E+00  0.000000E+00  0.000000E+00  0.000000E+00  0.000000E+00  0.000000E+00  0.000000E+00  0.000000E+00  0.000000E+00  0.000000E+00       Funtag_D
      59  5.408510E+05  5.039389E+05       36912.1       16.7610      0.116921       1.16546      0.198908      0.223164     0.0493706     0.0171082    0.00722971      0.988160       Opttag_D
      59       56.8143       2.38205       1.00000  0.000000E+00       1.00000  0.000000E+00      0.875250       62.0667       1.00000       16.5953  0.000000E+00       1.87483  0.000000E+00       1.87483  0.000000E+00  0.000000E+00      -3.74966       56.3253       2.16394        1       Scatag_D
      59       1469.47       5323.67  0.000000E+00  0.000000E+00       2347.62  0.000000E+00       2532.75       4978.00  0.000000E+00       10084.2       51.8786       10124.6 -1.000000E+10 -1.000000E+10 -1.000000E+10 -1.000000E+10 -1.000000E+10  0.000000E+00  0.000000E+00  0.000000E+00  0.000000E+00  0.000000E+00  0.000000E+00  0.000000E+00  0.000000E+00  0.000000E+00  0.000000E+00  0.000000E+00  0.000000E+00       Funtag_D
      60  5.407941E+05  5.038367E+05       36957.4       12.0440      0.119745       1.23111      0.198853      0.223123     0.0496283     0.0172126    0.00725123      0.988208       Opttag_D
      60       56.8565       2.38076       1.00000  0.000000E+00       1.00000  0.000000E+00      0.875184       62.0607       1.00000       16.6472  0.000000E+00       1.86790  0.000000E+00       1.86790  0.000000E+00  0.000000E+00      -3.73580       56.3253       2.16394        1       Scatag_D
      60       1478.23       5324.18  0.000000E+00  0.000000E+00       2351.33  0.000000E+00       2543.02       4997.22  0.000000E+00       10103.1       51.5701       10108.8 -1.000000E+10 -1.000000E+10 -1.000000E+10 -1.000000E+10 -1.000000E+10  0.000000E+00  0.000000E+00  0.000000E+00  0.000000E+00  0.000000E+00  0.000000E+00  0.000000E+00  0.000000E+00  0.000000E+00  0.000000E+00  0.000000E+00  0.000000E+00       Funtag_D
      61  5.407491E+05  5.038145E+05       36934.6       9.09575      0.120087       1.23967      0.198838      0.223111     0.0496842     0.0172559    0.00723743      0.987874       Opttag_D
      61       56.8609       2.38003       1.00000  0.000000E+00       1.00000  0.000000E+00      0.875172       62.0576       1.00000       16.6533  0.000000E+00       1.86762  0.000000E+00       1.86762  0.000000E+00  0.000000E+00      -3.73525       56.3253       2.16394        1       Scatag_D
      61       1472.61       5320.58  0.000000E+00  0.000000E+00       2351.31  0.000000E+00       2544.47       4989.79  0.000000E+00       10103.6       51.3941       10100.9 -1.000000E+10 -1.000000E+10 -1.000000E+10 -1.000000E+10 -1.000000E+10  0.000000E+00  0.000000E+00  0.000000E+00  0.000000E+00  0.000000E+00  0.000000E+00  0.000000E+00  0.000000E+00  0.000000E+00  0.000000E+00  0.000000E+00  0.000000E+00       Funtag_D
      62  5.406775E+05  5.037654E+05       36912.1       12.7975      0.120994       1.26193      0.198804      0.223079     0.0498081     0.0173646    0.00720796      0.987197       Opttag_D
      62       56.8724       2.37850       1.00000  0.000000E+00       1.00000  0.000000E+00      0.875138       62.0523       1.00000       16.6688  0.000000E+00       1.86689  0.000000E+00       1.86689  0.000000E+00  0.000000E+00      -3.73377       56.3253       2.16394        1       Scatag_D
      62       1460.64       5313.30  0.000000E+00  0.000000E+00       2351.18  0.000000E+00       2548.37       4999.93  0.000000E+00       10106.2       51.0633       10081.4 -1.000000E+10 -1.000000E+10 -1.000000E+10 -1.000000E+10 -1.000000E+10  0.000000E+00  0.000000E+00  0.000000E+00  0.000000E+00  0.000000E+00  0.000000E+00  0.000000E+00  0.000000E+00  0.000000E+00  0.000000E+00  0.000000E+00  0.000000E+00       Funtag_D
      63  5.406470E+05  5.037028E+05       36944.2       22.2605      0.122933       1.30576      0.198768      0.223046     0.0499659     0.0173965    0.00720417      0.986875       Opttag_D
      63       56.8984       2.37719       1.00000  0.000000E+00       1.00000  0.000000E+00      0.875083       62.0462       1.00000       16.7047  0.000000E+00       1.86480  0.000000E+00       1.86480  0.000000E+00  0.000000E+00      -3.72959       56.3253       2.16394        1       Scatag_D
      63       1459.10       5309.82  0.000000E+00  0.000000E+00       2352.78  0.000000E+00       2555.10       5038.60  0.000000E+00       10118.9       50.8220       10059.1 -1.000000E+10 -1.000000E+10 -1.000000E+10 -1.000000E+10 -1.000000E+10  0.000000E+00  0.000000E+00  0.000000E+00  0.000000E+00  0.000000E+00  0.000000E+00  0.000000E+00  0.000000E+00  0.000000E+00  0.000000E+00  0.000000E+00  0.000000E+00       Funtag_D
      64  5.405950E+05  5.037004E+05       36894.6       11.4678      0.122993       1.30715      0.198766      0.223045     0.0499718     0.0173941    0.00719772      0.986580       Opttag_D
      64       56.8987       2.37707       1.00000  0.000000E+00       1.00000  0.000000E+00      0.875079       62.0451       1.00000       16.7060  0.000000E+00       1.86468  0.000000E+00       1.86468  0.000000E+00  0.000000E+00      -3.72936       56.3253       2.16394        1       Scatag_D
      64       1456.49       5306.65  0.000000E+00  0.000000E+00       2352.66  0.000000E+00       2553.97       5000.70  0.000000E+00       10118.7       50.7819       10054.6 -1.000000E+10 -1.000000E+10 -1.000000E+10 -1.000000E+10 -1.000000E+10  0.000000E+00  0.000000E+00  0.000000E+00  0.000000E+00  0.000000E+00  0.000000E+00  0.000000E+00  0.000000E+00  0.000000E+00  0.000000E+00  0.000000E+00  0.000000E+00       Funtag_D
      65  5.405439E+05  5.036819E+05       36862.0       9.45892      0.123752       1.32362      0.198760      0.223049     0.0500185     0.0173569    0.00719471      0.986330       Opttag_D
      65       56.9081       2.37673       1.00000  0.000000E+00       1.00000  0.000000E+00      0.875054       62.0414       1.00000       16.7211  0.000000E+00       1.86362  0.000000E+00       1.86362  0.000000E+00  0.000000E+00      -3.72723       56.3253       2.16394        1       Scatag_D
      65       1455.28       5303.96  0.000000E+00  0.000000E+00       2353.33  0.000000E+00       2553.33       4982.15  0.000000E+00       10123.2       50.6399       10040.1 -1.000000E+10 -1.000000E+10 -1.000000E+10 -1.000000E+10 -1.000000E+10  0.000000E+00  0.000000E+00  0.000000E+00  0.000000E+00  0.000000E+00  0.000000E+00  0.000000E+00  0.000000E+00  0.000000E+00  0.000000E+00  0.000000E+00  0.000000E+00       Funtag_D
      66  5.405049E+05  5.036592E+05       36845.7       10.6129      0.124881       1.34757      0.198751      0.223060     0.0500757     0.0172883    0.00719575      0.986160       Opttag_D
      66       56.9222       2.37645       1.00000  0.000000E+00       1.00000  0.000000E+00      0.875020       62.0368       1.00000       16.7433  0.000000E+00       1.86211  0.000000E+00       1.86211  0.000000E+00  0.000000E+00      -3.72422       56.3253       2.16394        1       Scatag_D
      66       1455.70       5302.14  0.000000E+00  0.000000E+00       2354.67  0.000000E+00       2553.87       4976.57  0.000000E+00       10130.5       50.3692       10021.9 -1.000000E+10 -1.000000E+10 -1.000000E+10 -1.000000E+10 -1.000000E+10  0.000000E+00  0.000000E+00  0.000000E+00  0.000000E+00  0.000000E+00  0.000000E+00  0.000000E+00  0.000000E+00  0.000000E+00  0.000000E+00  0.000000E+00  0.000000E+00       Funtag_D
      67  5.404316E+05  5.036287E+05       36803.0       16.6379      0.127335       1.39716      0.198745      0.223130     0.0501528     0.0170633    0.00719332      0.985979       Opttag_D
      67       56.9518       2.37645       1.00000  0.000000E+00       1.00000  0.000000E+00      0.874943       62.0243       1.00000       16.7919  0.000000E+00       1.85886  0.000000E+00       1.85886  0.000000E+00  0.000000E+00      -3.71773       56.3253       2.16394        1       Scatag_D
      67       1454.71       5300.18  0.000000E+00  0.000000E+00       2358.49  0.000000E+00       2554.67       4963.65  0.000000E+00       10145.0       49.7066       9976.57 -1.000000E+10 -1.000000E+10 -1.000000E+10 -1.000000E+10 -1.000000E+10  0.000000E+00  0.000000E+00  0.000000E+00  0.000000E+00  0.000000E+00  0.000000E+00  0.000000E+00  0.000000E+00  0.000000E+00  0.000000E+00  0.000000E+00  0.000000E+00       Funtag_D
      68  5.404048E+05  5.036132E+05       36791.6       24.1383      0.130463       1.45626      0.198749      0.223289     0.0501918     0.0166946    0.00721527      0.986513       Opttag_D
      68       56.9882       2.37738       1.00000  0.000000E+00       1.00000  0.000000E+00      0.874848       62.0124       1.00000       16.8540  0.000000E+00       1.85451  0.000000E+00       1.85451  0.000000E+00  0.000000E+00      -3.70902       56.3253       2.16394        1       Scatag_D
      68       1463.60       5305.93  0.000000E+00  0.000000E+00       2365.15  0.000000E+00       2556.40       4964.11  0.000000E+00       10164.3       49.2760       9922.78 -1.000000E+10 -1.000000E+10 -1.000000E+10 -1.000000E+10 -1.000000E+10  0.000000E+00  0.000000E+00  0.000000E+00  0.000000E+00  0.000000E+00  0.000000E+00  0.000000E+00  0.000000E+00  0.000000E+00  0.000000E+00  0.000000E+00  0.000000E+00       Funtag_D
      69  5.403354E+05  5.036134E+05       36722.1       9.27484      0.129608       1.43943      0.198745      0.223259     0.0501913     0.0167588    0.00719925      0.986025       Opttag_D
      69       56.9778       2.37682       1.00000  0.000000E+00       1.00000  0.000000E+00      0.874865       62.0105       1.00000       16.8379  0.000000E+00       1.85542  0.000000E+00       1.85542  0.000000E+00  0.000000E+00      -3.71084       56.3253       2.16394        1       Scatag_D
      69       1457.11       5300.69  0.000000E+00  0.000000E+00       2363.36  0.000000E+00       2554.15       4917.60  0.000000E+00       10154.5       49.3989       9925.27 -1.000000E+10 -1.000000E+10 -1.000000E+10 -1.000000E+10 -1.000000E+10  0.000000E+00  0.000000E+00  0.000000E+00  0.000000E+00  0.000000E+00  0.000000E+00  0.000000E+00  0.000000E+00  0.000000E+00  0.000000E+00  0.000000E+00  0.000000E+00       Funtag_D
      70  5.403045E+05  5.036120E+05       36692.5       7.85182      0.129466       1.43547      0.198744      0.223267     0.0501947     0.0167432    0.00719595      0.985969       Opttag_D
      70       56.9755       2.37654       1.00000  0.000000E+00       1.00000  0.000000E+00      0.874861       62.0069       1.00000       16.8358  0.000000E+00       1.85536  0.000000E+00       1.85536  0.000000E+00  0.000000E+00      -3.71071       56.3253       2.16394        1       Scatag_D
      70       1455.78       5300.08  0.000000E+00  0.000000E+00       2363.23  0.000000E+00       2552.48       4905.77  0.000000E+00       10149.0       49.5359       9916.69 -1.000000E+10 -1.000000E+10 -1.000000E+10 -1.000000E+10 -1.000000E+10  0.000000E+00  0.000000E+00  0.000000E+00  0.000000E+00  0.000000E+00  0.000000E+00  0.000000E+00  0.000000E+00  0.000000E+00  0.000000E+00  0.000000E+00  0.000000E+00       Funtag_D
      71  5.402571E+05  5.036052E+05       36651.9       10.5480      0.130019       1.44280      0.198739      0.223318     0.0502120     0.0166332    0.00720372      0.986189       Opttag_D
      71       56.9809       2.37626       1.00000  0.000000E+00       1.00000  0.000000E+00      0.874829       62.0000       1.00000       16.8481  0.000000E+00       1.85404  0.000000E+00       1.85404  0.000000E+00  0.000000E+00      -3.70807       56.3253       2.16394        1       Scatag_D
      71       1458.92       5302.45  0.000000E+00  0.000000E+00       2364.76  0.000000E+00       2550.46       4892.58  0.000000E+00       10143.8       49.7665       9889.19 -1.000000E+10 -1.000000E+10 -1.000000E+10 -1.000000E+10 -1.000000E+10  0.000000E+00  0.000000E+00  0.000000E+00  0.000000E+00  0.000000E+00  0.000000E+00  0.000000E+00  0.000000E+00  0.000000E+00  0.000000E+00  0.000000E+00  0.000000E+00       Funtag_D
      72  5.402338E+05  5.035925E+05       36641.3       25.8884      0.131895       1.47061      0.198741      0.223445     0.0502439     0.0163778    0.00724343      0.987004       Opttag_D
      72       57.0007       2.37606       1.00000  0.000000E+00       1.00000  0.000000E+00      0.874748       61.9886       1.00000       16.8886  0.000000E+00       1.85179  0.000000E+00       1.85179  0.000000E+00  0.000000E+00      -3.70357       56.3253       2.16394        1       Scatag_D
      72       1475.05       5311.22  0.000000E+00  0.000000E+00       2369.45  0.000000E+00       2548.85       4905.44  0.000000E+00       10142.6       49.8089       9838.94 -1.000000E+10 -1.000000E+10 -1.000000E+10 -1.000000E+10 -1.000000E+10  0.000000E+00  0.000000E+00  0.000000E+00  0.000000E+00  0.000000E+00  0.000000E+00  0.000000E+00  0.000000E+00  0.000000E+00  0.000000E+00  0.000000E+00  0.000000E+00       Funtag_D
      73  5.401757E+05  5.035896E+05       36586.1       10.1808      0.132067       1.47279      0.198741      0.223451     0.0502512     0.0163650    0.00724106      0.986722       Opttag_D
      73       57.0025       2.37601       1.00000  0.000000E+00       1.00000  0.000000E+00      0.874740       61.9889       1.00000       16.8922  0.000000E+00       1.85149  0.000000E+00       1.85149  0.000000E+00  0.000000E+00      -3.70298       56.3253       2.16394        1       Scatag_D
      73       1474.09       5308.17  0.000000E+00  0.000000E+00       2369.78  0.000000E+00       2548.45       4860.24  0.000000E+00       10142.0       49.7661       9833.60 -1.000000E+10 -1.000000E+10 -1.000000E+10 -1.000000E+10 -1.000000E+10  0.000000E+00  0.000000E+00  0.000000E+00  0.000000E+00  0.000000E+00  0.000000E+00  0.000000E+00  0.000000E+00  0.000000E+00  0.000000E+00  0.000000E+00  0.000000E+00       Funtag_D
      74  5.401510E+05  5.035842E+05       36566.7       7.11958      0.132436       1.47823      0.198740      0.223462     0.0502648     0.0163519    0.00724176      0.986600       Opttag_D
      74       57.0066       2.37590       1.00000  0.000000E+00       1.00000  0.000000E+00      0.874725       61.9887       1.00000       16.9002  0.000000E+00       1.85131  0.000000E+00       1.85131  0.000000E+00  0.000000E+00      -3.70263       56.3253       2.16394        1       Scatag_D
      74       1474.37       5306.86  0.000000E+00  0.000000E+00       2370.48  0.000000E+00       2547.90       4849.24  0.000000E+00       10142.9       49.5882       9825.42 -1.000000E+10 -1.000000E+10 -1.000000E+10 -1.000000E+10 -1.000000E+10  0.000000E+00  0.000000E+00  0.000000E+00  0.000000E+00  0.000000E+00  0.000000E+00  0.000000E+00  0.000000E+00  0.000000E+00  0.000000E+00  0.000000E+00  0.000000E+00       Funtag_D
      75  5.401235E+05  5.035776E+05       36546.0       8.58348      0.133152       1.48794      0.198740      0.223497     0.0502816     0.0163093    0.00724614      0.986574       Opttag_D
      75       57.0142       2.37576       1.00000  0.000000E+00       1.00000  0.000000E+00      0.874695       61.9861       1.00000       16.9162  0.000000E+00       1.85108  0.000000E+00       1.85108  0.000000E+00  0.000000E+00      -3.70216       56.3253       2.16394        1       Scatag_D
      75       1476.16       5306.59  0.000000E+00  0.000000E+00       2371.93  0.000000E+00       2546.84       4842.06  0.000000E+00       10143.7       49.2969       9809.40 -1.000000E+10 -1.000000E+10 -1.000000E+10 -1.000000E+10 -1.000000E+10  0.000000E+00  0.000000E+00  0.000000E+00  0.000000E+00  0.000000E+00  0.000000E+00  0.000000E+00  0.000000E+00  0.000000E+00  0.000000E+00  0.000000E+00  0.000000E+00       Funtag_D
      76  5.400835E+05  5.035688E+05       36514.7       10.5169      0.134102       1.49915      0.198743      0.223537     0.0503037     0.0162876    0.00724021      0.986504       Opttag_D
      76       57.0241       2.37546       1.00000  0.000000E+00       1.00000  0.000000E+00      0.874652       61.9790       1.00000       16.9389  0.000000E+00       1.85109  0.000000E+00       1.85109  0.000000E+00  0.000000E+00      -3.70218       56.3253       2.16394        1       Scatag_D
      76       1473.74       5305.84  0.000000E+00  0.000000E+00       2373.71  0.000000E+00       2545.27       4836.21  0.000000E+00       10144.3       48.8859       9786.79 -1.000000E+10 -1.000000E+10 -1.000000E+10 -1.000000E+10 -1.000000E+10  0.000000E+00  0.000000E+00  0.000000E+00  0.000000E+00  0.000000E+00  0.000000E+00  0.000000E+00  0.000000E+00  0.000000E+00  0.000000E+00  0.000000E+00  0.000000E+00       Funtag_D

 An atom has moved more than     1.50 A from the initial/last
 position - if this was an accepted trial point, probably need to update the
 non-bonded contact list.


 An atom has moved more than     1.50 A from the initial/last
 position - if this was an accepted trial point, probably need to update the
 non-bonded contact list.

      77  5.400572E+05  5.035618E+05       36495.4       12.8959      0.135044       1.50923      0.198746      0.223586     0.0503214     0.0162443    0.00723583      0.986601       Opttag_D
      77       57.0337       2.37521       1.00000  0.000000E+00       1.00000  0.000000E+00      0.874610       61.9726       1.00000       16.9615  0.000000E+00       1.85103  0.000000E+00       1.85103  0.000000E+00  0.000000E+00      -3.70206       56.3253       2.16394        2       Scatag_D
      77       1471.96       5306.88  0.000000E+00  0.000000E+00       2375.58  0.000000E+00       2544.28       4834.74  0.000000E+00       10145.1       48.5783       9768.36 -1.000000E+10 -1.000000E+10 -1.000000E+10 -1.000000E+10 -1.000000E+10  0.000000E+00  0.000000E+00  0.000000E+00  0.000000E+00  0.000000E+00  0.000000E+00  0.000000E+00  0.000000E+00  0.000000E+00  0.000000E+00  0.000000E+00  0.000000E+00       Funtag_D

 An atom has moved more than     1.50 A from the initial/last
 position - need to update the non-bonded contact list.


 ############################################################################################################
 ###################################################### setting up contact terms
 ############################################################################################################

      78  5.400254E+05  5.035551E+05       36470.3       7.62500      0.135541       1.51285      0.198747      0.223603     0.0503381     0.0162559    0.00722438      0.986522       Opttag_D
      78       57.0384       2.37485       1.00000  0.000000E+00       1.00000  0.000000E+00      0.874585       61.9655       1.00000       16.9746  0.000000E+00       1.85105  0.000000E+00       1.85105  0.000000E+00  0.000000E+00      -3.70211       56.3253       2.16394        1       Scatag_D
      78       1467.30       5306.02  0.000000E+00  0.000000E+00       2376.44  0.000000E+00       2543.88       4826.38  0.000000E+00       10144.8       48.4112       9757.08 -1.000000E+10 -1.000000E+10 -1.000000E+10 -1.000000E+10 -1.000000E+10  0.000000E+00  0.000000E+00  0.000000E+00  0.000000E+00  0.000000E+00  0.000000E+00  0.000000E+00  0.000000E+00  0.000000E+00  0.000000E+00  0.000000E+00  0.000000E+00       Funtag_D
      79  5.399974E+05  5.035430E+05       36454.4       7.04282      0.135896       1.51339      0.198743      0.223618     0.0503686     0.0162693    0.00721012      0.986410       Opttag_D
      79       57.0413       2.37417       1.00000  0.000000E+00       1.00000  0.000000E+00      0.874565       61.9578       1.00000       16.9850  0.000000E+00       1.85090  0.000000E+00       1.85090  0.000000E+00  0.000000E+00      -3.70180       56.3253       2.16394        1       Scatag_D
      79       1461.52       5304.82  0.000000E+00  0.000000E+00       2377.03  0.000000E+00       2543.88       4828.27  0.000000E+00       10143.4       48.3613       9747.11 -1.000000E+10 -1.000000E+10 -1.000000E+10 -1.000000E+10 -1.000000E+10  0.000000E+00  0.000000E+00  0.000000E+00  0.000000E+00  0.000000E+00  0.000000E+00  0.000000E+00  0.000000E+00  0.000000E+00  0.000000E+00  0.000000E+00  0.000000E+00       Funtag_D
      80  5.399707E+05  5.035203E+05       36450.3       10.1855      0.136411       1.51365      0.198733      0.223644     0.0504259     0.0162754    0.00720084      0.986324       Opttag_D
      80       57.0453       2.37304       1.00000  0.000000E+00       1.00000  0.000000E+00      0.874538       61.9492       1.00000       16.9990  0.000000E+00       1.85022  0.000000E+00       1.85022  0.000000E+00  0.000000E+00      -3.70045       56.3253       2.16394        1       Scatag_D
      80       1457.76       5303.90  0.000000E+00  0.000000E+00       2378.05  0.000000E+00       2544.68       4839.84  0.000000E+00       10141.6       48.3211       9736.23 -1.000000E+10 -1.000000E+10 -1.000000E+10 -1.000000E+10 -1.000000E+10  0.000000E+00  0.000000E+00  0.000000E+00  0.000000E+00  0.000000E+00  0.000000E+00  0.000000E+00  0.000000E+00  0.000000E+00  0.000000E+00  0.000000E+00  0.000000E+00       Funtag_D
      81  5.399446E+05  5.034926E+05       36452.0       8.56887      0.136954       1.51335      0.198722      0.223682     0.0504957     0.0162395    0.00720263      0.986140       Opttag_D
      81       57.0494       2.37172       1.00000  0.000000E+00       1.00000  0.000000E+00      0.874511       61.9414       1.00000       17.0130  0.000000E+00       1.84945  0.000000E+00       1.84945  0.000000E+00  0.000000E+00      -3.69891       56.3253       2.16394        1       Scatag_D
      81       1458.48       5301.91  0.000000E+00  0.000000E+00       2379.23  0.000000E+00       2546.24       4849.85  0.000000E+00       10140.1       48.2782       9727.89 -1.000000E+10 -1.000000E+10 -1.000000E+10 -1.000000E+10 -1.000000E+10  0.000000E+00  0.000000E+00  0.000000E+00  0.000000E+00  0.000000E+00  0.000000E+00  0.000000E+00  0.000000E+00  0.000000E+00  0.000000E+00  0.000000E+00  0.000000E+00       Funtag_D
      82  5.399238E+05  5.034731E+05       36450.6       6.63742      0.137137       1.51198      0.198714      0.223697     0.0505449     0.0162142    0.00720872      0.985883       Opttag_D
      82       57.0505       2.37085       1.00000  0.000000E+00       1.00000  0.000000E+00      0.874502       61.9388       1.00000       17.0172  0.000000E+00       1.84902  0.000000E+00       1.84902  0.000000E+00  0.000000E+00      -3.69805       56.3253       2.16394        1       Scatag_D
      82       1460.95       5299.16  0.000000E+00  0.000000E+00       2379.72  0.000000E+00       2547.20       4852.53  0.000000E+00       10139.0       48.2172       9723.86 -1.000000E+10 -1.000000E+10 -1.000000E+10 -1.000000E+10 -1.000000E+10  0.000000E+00  0.000000E+00  0.000000E+00  0.000000E+00  0.000000E+00  0.000000E+00  0.000000E+00  0.000000E+00  0.000000E+00  0.000000E+00  0.000000E+00  0.000000E+00       Funtag_D
      83  5.398948E+05  5.034351E+05       36459.7       7.97214      0.137559       1.50879      0.198695      0.223726     0.0506407     0.0161702    0.00722368      0.985453       Opttag_D
      83       57.0536       2.36922       1.00000  0.000000E+00       1.00000  0.000000E+00      0.874483       61.9337       1.00000       17.0257  0.000000E+00       1.84821  0.000000E+00       1.84821  0.000000E+00  0.000000E+00      -3.69642       56.3253       2.16394        1       Scatag_D
      83       1467.02       5294.54  0.000000E+00  0.000000E+00       2380.78  0.000000E+00       2549.46       4866.65  0.000000E+00       10137.0       48.0090       9716.23 -1.000000E+10 -1.000000E+10 -1.000000E+10 -1.000000E+10 -1.000000E+10  0.000000E+00  0.000000E+00  0.000000E+00  0.000000E+00  0.000000E+00  0.000000E+00  0.000000E+00  0.000000E+00  0.000000E+00  0.000000E+00  0.000000E+00  0.000000E+00       Funtag_D
      84  5.398740E+05  5.034028E+05       36471.3       11.5390      0.138095       1.50466      0.198682      0.223766     0.0507222     0.0161192    0.00723043      0.985297       Opttag_D
      84       57.0577       2.36781       1.00000  0.000000E+00       1.00000  0.000000E+00      0.874460       61.9265       1.00000       17.0371  0.000000E+00       1.84764  0.000000E+00       1.84764  0.000000E+00  0.000000E+00      -3.69528       56.3253       2.16394        1       Scatag_D
      84       1469.76       5292.86  0.000000E+00  0.000000E+00       2381.94  0.000000E+00       2551.56       4883.29  0.000000E+00       10135.3       47.7523       9708.76 -1.000000E+10 -1.000000E+10 -1.000000E+10 -1.000000E+10 -1.000000E+10  0.000000E+00  0.000000E+00  0.000000E+00  0.000000E+00  0.000000E+00  0.000000E+00  0.000000E+00  0.000000E+00  0.000000E+00  0.000000E+00  0.000000E+00  0.000000E+00       Funtag_D
      85  5.398507E+05  5.033910E+05       36459.7       7.41604      0.138404       1.50201      0.198681      0.223784     0.0507518     0.0161101    0.00722757      0.985048       Opttag_D
      85       57.0603       2.36730       1.00000  0.000000E+00       1.00000  0.000000E+00      0.874446       61.9222       1.00000       17.0436  0.000000E+00       1.84759  0.000000E+00       1.84759  0.000000E+00  0.000000E+00      -3.69517       56.3253       2.16394        1       Scatag_D
      85       1468.60       5290.18  0.000000E+00  0.000000E+00       2382.53  0.000000E+00       2552.00       4880.58  0.000000E+00       10134.5       47.5924       9703.72 -1.000000E+10 -1.000000E+10 -1.000000E+10 -1.000000E+10 -1.000000E+10  0.000000E+00  0.000000E+00  0.000000E+00  0.000000E+00  0.000000E+00  0.000000E+00  0.000000E+00  0.000000E+00  0.000000E+00  0.000000E+00  0.000000E+00  0.000000E+00       Funtag_D
      86  5.398219E+05  5.033791E+05       36442.7       6.29392      0.139084       1.49611      0.198678      0.223819     0.0507820     0.0160948    0.00721678      0.984926       Opttag_D
      86       57.0659       2.36679       1.00000  0.000000E+00       1.00000  0.000000E+00      0.874418       61.9139       1.00000       17.0582  0.000000E+00       1.84758  0.000000E+00       1.84758  0.000000E+00  0.000000E+00      -3.69516       56.3253       2.16394        1       Scatag_D
      86       1464.22       5288.88  0.000000E+00  0.000000E+00       2383.73  0.000000E+00       2551.31       4881.81  0.000000E+00       10132.9       47.3269       9692.53 -1.000000E+10 -1.000000E+10 -1.000000E+10 -1.000000E+10 -1.000000E+10  0.000000E+00  0.000000E+00  0.000000E+00  0.000000E+00  0.000000E+00  0.000000E+00  0.000000E+00  0.000000E+00  0.000000E+00  0.000000E+00  0.000000E+00  0.000000E+00       Funtag_D
      87  5.398042E+05  5.033750E+05       36429.2       8.56806      0.139616       1.49030      0.198686      0.223858     0.0507924     0.0160511    0.00720911      0.984918       Opttag_D
      87       57.0701       2.36658       1.00000  0.000000E+00       1.00000  0.000000E+00      0.874395       61.9090       1.00000       17.0702  0.000000E+00       1.84750  0.000000E+00       1.84750  0.000000E+00  0.000000E+00      -3.69500       56.3253       2.16394        1       Scatag_D
      87       1461.11       5288.78  0.000000E+00  0.000000E+00       2384.63  0.000000E+00       2549.38       4884.02  0.000000E+00       10131.4       47.1604       9682.72 -1.000000E+10 -1.000000E+10 -1.000000E+10 -1.000000E+10 -1.000000E+10  0.000000E+00  0.000000E+00  0.000000E+00  0.000000E+00  0.000000E+00  0.000000E+00  0.000000E+00  0.000000E+00  0.000000E+00  0.000000E+00  0.000000E+00  0.000000E+00       Funtag_D
      88  5.397780E+05  5.033754E+05       36402.6       7.50970      0.140347       1.48146      0.198701      0.223908     0.0507915     0.0159824    0.00719670      0.984884       Opttag_D
      88       57.0758       2.36653       1.00000  0.000000E+00       1.00000  0.000000E+00      0.874363       61.9023       1.00000       17.0870  0.000000E+00       1.84758  0.000000E+00       1.84758  0.000000E+00  0.000000E+00      -3.69517       56.3253       2.16394        1       Scatag_D
      88       1456.08       5288.42  0.000000E+00  0.000000E+00       2385.80  0.000000E+00       2545.83       4882.03  0.000000E+00       10129.1       46.9893       9668.33 -1.000000E+10 -1.000000E+10 -1.000000E+10 -1.000000E+10 -1.000000E+10  0.000000E+00  0.000000E+00  0.000000E+00  0.000000E+00  0.000000E+00  0.000000E+00  0.000000E+00  0.000000E+00  0.000000E+00  0.000000E+00  0.000000E+00  0.000000E+00       Funtag_D
      89  5.397546E+05  5.033870E+05       36367.7       15.4334      0.141682       1.46267      0.198735      0.224005     0.0507621     0.0158147    0.00718139      0.985180       Opttag_D
      89       57.0857       2.36680       1.00000  0.000000E+00       1.00000  0.000000E+00      0.874307       61.8889       1.00000       17.1189  0.000000E+00       1.84770  0.000000E+00       1.84770  0.000000E+00  0.000000E+00      -3.69540       56.3253       2.16394        1       Scatag_D
      89       1449.89       5291.61  0.000000E+00  0.000000E+00       2387.77  0.000000E+00       2539.33       4887.29  0.000000E+00       10124.8       46.6686       9640.37 -1.000000E+10 -1.000000E+10 -1.000000E+10 -1.000000E+10 -1.000000E+10  0.000000E+00  0.000000E+00  0.000000E+00  0.000000E+00  0.000000E+00  0.000000E+00  0.000000E+00  0.000000E+00  0.000000E+00  0.000000E+00  0.000000E+00  0.000000E+00       Funtag_D
      90  5.397224E+05  5.033877E+05       36334.7       7.12234      0.141936       1.45695      0.198743      0.224033     0.0507602     0.0157492    0.00717502      0.984763       Opttag_D
      90       57.0874       2.36670       1.00000  0.000000E+00       1.00000  0.000000E+00      0.874294       61.8836       1.00000       17.1268  0.000000E+00       1.84790  0.000000E+00       1.84790  0.000000E+00  0.000000E+00      -3.69581       56.3253       2.16394        1       Scatag_D
      90       1447.32       5287.12  0.000000E+00  0.000000E+00       2387.93  0.000000E+00       2537.55       4874.12  0.000000E+00       10122.6       46.5985       9631.49 -1.000000E+10 -1.000000E+10 -1.000000E+10 -1.000000E+10 -1.000000E+10  0.000000E+00  0.000000E+00  0.000000E+00  0.000000E+00  0.000000E+00  0.000000E+00  0.000000E+00  0.000000E+00  0.000000E+00  0.000000E+00  0.000000E+00  0.000000E+00       Funtag_D
      91  5.397033E+05  5.033851E+05       36318.1       6.47117      0.141957       1.45306      0.198744      0.224039     0.0507667     0.0157193    0.00717314      0.984597       Opttag_D
      91       57.0874       2.36644       1.00000  0.000000E+00       1.00000  0.000000E+00      0.874291       61.8774       1.00000       17.1296  0.000000E+00       1.84813  0.000000E+00       1.84813  0.000000E+00  0.000000E+00      -3.69626       56.3253       2.16394        1       Scatag_D
      91       1446.56       5285.34  0.000000E+00  0.000000E+00       2387.61  0.000000E+00       2536.85       4869.91  0.000000E+00       10120.1       46.5385       9625.26 -1.000000E+10 -1.000000E+10 -1.000000E+10 -1.000000E+10 -1.000000E+10  0.000000E+00  0.000000E+00  0.000000E+00  0.000000E+00  0.000000E+00  0.000000E+00  0.000000E+00  0.000000E+00  0.000000E+00  0.000000E+00  0.000000E+00  0.000000E+00       Funtag_D
      92  5.396751E+05  5.033821E+05       36293.0       8.09953      0.142450       1.43994      0.198756      0.224072     0.0507745     0.0156206    0.00717108      0.984446       Opttag_D
      92       57.0905       2.36594       1.00000  0.000000E+00       1.00000  0.000000E+00      0.874270       61.8596       1.00000       17.1454  0.000000E+00       1.84859  0.000000E+00       1.84859  0.000000E+00  0.000000E+00      -3.69718       56.3253       2.16394        1       Scatag_D
      92       1445.73       5283.72  0.000000E+00  0.000000E+00       2387.70  0.000000E+00       2536.04       4868.74  0.000000E+00       10115.5       46.4237       9609.21 -1.000000E+10 -1.000000E+10 -1.000000E+10 -1.000000E+10 -1.000000E+10  0.000000E+00  0.000000E+00  0.000000E+00  0.000000E+00  0.000000E+00  0.000000E+00  0.000000E+00  0.000000E+00  0.000000E+00  0.000000E+00  0.000000E+00  0.000000E+00       Funtag_D
      93  5.396680E+05  5.033770E+05       36291.0       20.2285      0.143615       1.41313      0.198778      0.224131     0.0507873     0.0154217    0.00717174      0.984587       Opttag_D
      93       57.0971       2.36500       1.00000  0.000000E+00       1.00000  0.000000E+00      0.874228       61.8255       1.00000       17.1789  0.000000E+00       1.84981  0.000000E+00       1.84981  0.000000E+00  0.000000E+00      -3.69961       56.3253       2.16394        1       Scatag_D
      93       1446.00       5285.23  0.000000E+00  0.000000E+00       2388.31  0.000000E+00       2537.05       4892.38  0.000000E+00       10109.4       46.3195       9586.33 -1.000000E+10 -1.000000E+10 -1.000000E+10 -1.000000E+10 -1.000000E+10  0.000000E+00  0.000000E+00  0.000000E+00  0.000000E+00  0.000000E+00  0.000000E+00  0.000000E+00  0.000000E+00  0.000000E+00  0.000000E+00  0.000000E+00  0.000000E+00       Funtag_D
      94  5.396303E+05  5.033735E+05       36256.8       7.31672      0.143602       1.41349      0.198774      0.224124     0.0507961     0.0154404    0.00716798      0.984025       Opttag_D
      94       57.0969       2.36488       1.00000  0.000000E+00       1.00000  0.000000E+00      0.874230       61.8251       1.00000       17.1780  0.000000E+00       1.84969  0.000000E+00       1.84969  0.000000E+00  0.000000E+00      -3.69938       56.3253       2.16394        1       Scatag_D
      94       1444.48       5279.20  0.000000E+00  0.000000E+00       2388.33  0.000000E+00       2537.37       4866.04  0.000000E+00       10109.7       46.3263       9585.40 -1.000000E+10 -1.000000E+10 -1.000000E+10 -1.000000E+10 -1.000000E+10  0.000000E+00  0.000000E+00  0.000000E+00  0.000000E+00  0.000000E+00  0.000000E+00  0.000000E+00  0.000000E+00  0.000000E+00  0.000000E+00  0.000000E+00  0.000000E+00       Funtag_D
      95  5.396156E+05  5.033693E+05       36246.3       5.34890      0.143833       1.40925      0.198774      0.224127     0.0508066     0.0154202    0.00716636      0.983881       Opttag_D
      95       57.0979       2.36463       1.00000  0.000000E+00       1.00000  0.000000E+00      0.874223       61.8203       1.00000       17.1832  0.000000E+00       1.84977  0.000000E+00       1.84977  0.000000E+00  0.000000E+00      -3.69954       56.3253       2.16394        1       Scatag_D
      95       1443.83       5277.65  0.000000E+00  0.000000E+00       2388.60  0.000000E+00       2537.83       4861.20  0.000000E+00       10109.4       46.3320       9581.37 -1.000000E+10 -1.000000E+10 -1.000000E+10 -1.000000E+10 -1.000000E+10  0.000000E+00  0.000000E+00  0.000000E+00  0.000000E+00  0.000000E+00  0.000000E+00  0.000000E+00  0.000000E+00  0.000000E+00  0.000000E+00  0.000000E+00  0.000000E+00       Funtag_D
      96  5.395959E+05  5.033607E+05       36235.3       6.59556      0.144417       1.39710      0.198778      0.224146     0.0508284     0.0153394    0.00716895      0.983694       Opttag_D
      96       57.1005       2.36403       1.00000  0.000000E+00       1.00000  0.000000E+00      0.874204       61.8077       1.00000       17.1974  0.000000E+00       1.85015  0.000000E+00       1.85015  0.000000E+00  0.000000E+00      -3.70030       56.3253       2.16394        1       Scatag_D
      96       1444.87       5275.65  0.000000E+00  0.000000E+00       2389.18  0.000000E+00       2538.67       4859.91  0.000000E+00       10108.3       46.3293       9572.38 -1.000000E+10 -1.000000E+10 -1.000000E+10 -1.000000E+10 -1.000000E+10  0.000000E+00  0.000000E+00  0.000000E+00  0.000000E+00  0.000000E+00  0.000000E+00  0.000000E+00  0.000000E+00  0.000000E+00  0.000000E+00  0.000000E+00  0.000000E+00       Funtag_D
      97  5.395679E+05  5.033442E+05       36223.7       9.15203      0.145324       1.37656      0.198782      0.224190     0.0508699     0.0151611    0.00718119      0.983414       Opttag_D
      97       57.1041       2.36298       1.00000  0.000000E+00       1.00000  0.000000E+00      0.874173       61.7899       1.00000       17.2196  0.000000E+00       1.85063  0.000000E+00       1.85063  0.000000E+00  0.000000E+00      -3.70126       56.3253       2.16394        1       Scatag_D
      97       1449.81       5272.64  0.000000E+00  0.000000E+00       2390.12  0.000000E+00       2539.32       4860.67  0.000000E+00       10106.4       46.2274       9558.50 -1.000000E+10 -1.000000E+10 -1.000000E+10 -1.000000E+10 -1.000000E+10  0.000000E+00  0.000000E+00  0.000000E+00  0.000000E+00  0.000000E+00  0.000000E+00  0.000000E+00  0.000000E+00  0.000000E+00  0.000000E+00  0.000000E+00  0.000000E+00       Funtag_D
      98  5.395477E+05  5.033181E+05       36229.6       13.1096      0.147078       1.34761      0.198790      0.224275     0.0509357     0.0148186    0.00721648      0.983427       Opttag_D
      98       57.1112       2.36119       1.00000  0.000000E+00       1.00000  0.000000E+00      0.874113       61.7588       1.00000       17.2628  0.000000E+00       1.85195  0.000000E+00       1.85195  0.000000E+00  0.000000E+00      -3.70390       56.3253       2.16394        1       Scatag_D
      98       1464.10       5272.79  0.000000E+00  0.000000E+00       2392.02  0.000000E+00       2541.21       4869.72  0.000000E+00       10105.5       46.1325       9538.08 -1.000000E+10 -1.000000E+10 -1.000000E+10 -1.000000E+10 -1.000000E+10  0.000000E+00  0.000000E+00  0.000000E+00  0.000000E+00  0.000000E+00  0.000000E+00  0.000000E+00  0.000000E+00  0.000000E+00  0.000000E+00  0.000000E+00  0.000000E+00       Funtag_D
      99  5.395169E+05  5.033147E+05       36202.2       6.20663      0.147002       1.34387      0.198786      0.224279     0.0509445     0.0148062    0.00721030      0.983035       Opttag_D
      99       57.1109       2.36104       1.00000  0.000000E+00       1.00000  0.000000E+00      0.874113       61.7592       1.00000       17.2618  0.000000E+00       1.85185  0.000000E+00       1.85185  0.000000E+00  0.000000E+00      -3.70371       56.3253       2.16394        1       Scatag_D
      99       1461.59       5268.59  0.000000E+00  0.000000E+00       2391.90  0.000000E+00       2540.29       4857.33  0.000000E+00       10104.6       45.9941       9531.93 -1.000000E+10 -1.000000E+10 -1.000000E+10 -1.000000E+10 -1.000000E+10  0.000000E+00  0.000000E+00  0.000000E+00  0.000000E+00  0.000000E+00  0.000000E+00  0.000000E+00  0.000000E+00  0.000000E+00  0.000000E+00  0.000000E+00  0.000000E+00       Funtag_D

   MaxLik Scaling Cycle 0106

   Wilson plot

   Statistics in resolution bins after refinement cycle 0003
```


|  |  |
| --- | --- |
|  | - Rxpct factors plot- Rxpct factors table- <Log-Lik Gain>- Ln Sigma A- Correlation Coefficients |


```
   Iters     Tot_Funct    Xray_Funct    Geom_Funct          Grms     rmsD2init     maxD2init             R         Rfree           LLG       LLGfree     rms(bond)    rms(angle)
   Iters             K   B_IMPF_FRAG        K_MISS   B_TEMP_MISS   K_IMPF_MISS   B_IMPF_MISS        K_SOLV   B_TEMP_SOLV   K_IMPF_SOLV   B_IMPF_SOLV        B_TEMP     B_TEMP_11     B_TEMP_12     B_TEMP_22     B_TEMP_13     B_TEMP_23     B_TEMP_33      K_WILSON      B_WILSON      FGCYCLES
   Iters          BOND         ANGLE      IMPROPER       TORSION    SINTORSION      TRIGONAL        PLANAR  BCORRELATION       CONTACT     IDEALDIST        CHIRAL    SIMILARITY       OCCSSUM  UTILDISTANCE     UTILANGLE   UTILTORSION        RHOFIT        ADP-01        ADP-02        ADP-03        ADP-04        ADP-05        ADP-06        ADP-07        ADP-08        ADP-09        ADP-10      RHOFITPC            QM
     100  5.394925E+05  5.033083E+05       36184.2       7.15678      0.147089       1.34032      0.198785      0.224293     0.0509605     0.0147612    0.00721122      0.982877       Opttag_D
     100       57.1114       2.36067       1.00000  0.000000E+00       1.00000  0.000000E+00      0.874104       61.7562       1.00000       17.2656  0.000000E+00       1.85202  0.000000E+00       1.85202  0.000000E+00  0.000000E+00      -3.70404       56.3253       2.16394        1       Scatag_D
     100       1461.96       5266.89  0.000000E+00  0.000000E+00       2391.95  0.000000E+00       2539.19       4854.73  0.000000E+00       10103.4       45.8008       9520.25 -1.000000E+10 -1.000000E+10 -1.000000E+10 -1.000000E+10 -1.000000E+10  0.000000E+00  0.000000E+00  0.000000E+00  0.000000E+00  0.000000E+00  0.000000E+00  0.000000E+00  0.000000E+00  0.000000E+00  0.000000E+00  0.000000E+00  0.000000E+00       Funtag_D
   Geometry Restraint Deviations SCREEN Information

 
 No -bcorrel_zeroocchydrogens_factor flag found - keeping default: will set B for OCC=0 hydrogens to    1.200 times heavy-atom B


 TLS information. Total number of groups:     2


 Group   Natoms    FixRB   FixTLS   Group Tag    Group Spec
     1     2770      Y        Y     tls1    { A|* }
     2     2774      Y        Y     tls2    { B|* }
 
NOTE BUSTER_TLS_SET tls1 { A|* }
NOTE BUSTER_TLS_T   tls1     -2.09805E-01 -1.15711E-01  3.73362E-01 -2.40920E-02  4.96139E-02  2.21940E-02
NOTE BUSTER_TLS_L   tls1      1.24514E+00  1.05226E+00  6.66191E-01  1.62361E-01 -3.88521E-01  3.54587E-01
NOTE BUSTER_TLS_S   tls1      2.06506E-01 -1.53727E-01  1.93700E-01 -1.05534E-01 -6.67227E-02 -9.37109E-02 -4.76199E-02 -4.26923E-02
NOTE BUSTER_TLS_O   tls1     -2.33748E+01 -8.36624E+01  1.14308E+01
NOTE BUSTER_TLS_FIX tls1 ALL
 
NOTE BUSTER_TLS_SET tls2 { B|* }
NOTE BUSTER_TLS_T   tls2     -2.19975E-01 -1.58351E-01  4.94077E-01  1.93068E-02  4.62273E-02  2.44113E-03
NOTE BUSTER_TLS_L   tls2      1.30038E+00  8.42752E-01  9.70856E-01  6.65317E-01 -8.38129E-01 -5.84650E-01
NOTE BUSTER_TLS_S   tls2     -2.69202E-02  1.09150E-01 -8.14403E-02  6.77598E-02  1.92600E-01  1.97964E-02 -6.29584E-02  2.14661E-02
NOTE BUSTER_TLS_O   tls2      2.20570E+01 -7.19562E+01  3.49479E+00
NOTE BUSTER_TLS_FIX tls2 ALL

 For final position, Geometry function summary
     term                           number     weight       rms        GooF      function     fn/numb
     ================================================================================================
     BOND bond lengths (angs)       5660       2.000      0.0072       0.359      1461.96       0.258
     ANGL bond angles (degs)        7681       2.000        0.98       0.586      5266.89       0.686
     TORS split-harmonic tors       1270       0.000       17.51       1.102         0.00       0.000
     SINTOR sinusoidal tors         1270       2.000       17.51                  2391.95       1.883
     PLAN general plane (angs)       964       5.000      0.0145       0.726      2539.19       2.634
     BCORrelations (angs^2)         5660      10.000       0.821       0.293      4854.73       0.858
     CONT (bad contacts)               0       5.000                                 0.00
     IDEAL(ideal-dist contact)      5067       4.000                             10103.44
     CHIRAL (gelly semiharmon)       740       5.000                                45.80
     SIM similarity restraint for NCS and/or TARGET                               9520.25
     OCCSUM restraint on sum of occupanies                                           0.00       as none defined
     DISTAN utility restraints interatomic distance                                  0.00       as none defined
     UTILANGLE utility angle restraints                                              0.00       as none defined
     UTILTOR utility restraints on torsion angles                                    0.00       as none defined
     Peptide omega torsion angles                           3.01 degs - monitor only
 
 WARNING: Have |delta/sigma| deviations > 5.0 sigma. Number of outliers for each term: 
 WARNING:         4 bond angles.  Worst is     5.4 sigs   135.28 degs A|28:C.B(ASN)=A|29:N(ASN)=A|29:CA(ASN)

 Weighted rms      0.0072         0.983  (temporary rms bond and angle for BUSTER to pick up!)
 Weighted intra-residual rms(bond) for StdProtein (excluding H)      0.0070
 Weighted intra-residual rms(bond) for Buffer     (excluding H)      0.0034
 Outputing geometry progress graph in plotmtv format to file:Geom_Res.mtv

 ############### Running final geometry/screen analysis (screen_final.txt)

 s/r gelly normal termination
```

Statistics and scale factors vs. cycle number
```

|  |  |  |
| --- | --- | --- |
|  | - Rxpct factors- <Log-Lik Gain>- GEOM Residuals- Ln Sigma_A_ | - Scale Factors- B Factors- Imperfection Factors |

```
   Final Geometry Information


---


### 2. Final results

Statistics and scale factors vs. cycle number
```

|  |  |  |
| --- | --- | --- |
|  | - Rxpct factors- <Log-Lik Gain>- GEOM Residuals- Ln Sigma_A_ | - Scale Factors- B Factors- Imperfection Factors |

```
   Average Figure of Merit
   Structure Factor Amplitudes


 # normal termination

  Program stopped.
```

closesok message: time spent in tree = 1654.15 (user) 37.16 (sys)

---

# Normal termination

```
 Resources usage summary:
     Time spent in client ...................  1654.16 (user)       37.18 (system)
     Time spent in scripts (    27 calls) ...     2.46 (user)        0.18 (system)
     Time spent in server ...................     0.00 (user)        0.00 (system)

     Max resident memory used (kb) .......... 444392
     Number of page faults .................. 0 (major)    889016 (minor)
     Number of context switches ............. 70862 (niced)    6217 (other)
```
